# Novel Chlorin e6-Curcumin Derivatives as a Potential Photosensitizer: Synthesis, Characterization, and Anticancer Activity

**DOI:** 10.3390/pharmaceutics15061577

**Published:** 2023-05-23

**Authors:** Til Bahadur Thapa Magar, Jusuk Lee, Ji Hoon Lee, Juhee Jeon, Pallavi Gurung, Junmo Lim, Yong-Wan Kim

**Affiliations:** 1 Dongsung Cancer Center, Dongsung Biopharmaceutical, Daegu 41061, Republic of Koreaijm15@ds-pharm.co.kr (J.L.); 2 A&J Science Co., Ltd., Daegu 41061, Republic of Korea; 3 New Drug Development Center, Daegu-Gyeongbuk Medical Innovation Foundation, Daegu 41061, Republic of Korea

**Keywords:** chlorin e6, curcumin, photosensitizer, photodynamic therapy, pancreatic carcinoma, melanoma

## Abstract

Novel series of chlorin e6-curcumin derivatives were designed and synthesized. All the synthesized compounds **16**, **17**, **18**, and **19** were tested for their photodynamic treatment (PDT) efficacy against human pancreatic cancer cell lines: AsPC-1, MIA-PaCa-2, and PANC-1. The cellular uptake study was performed in the aforementioned cell lines using fluorescence-activated cell sorting (FACS). **17**, among the synthesized compounds with IC_50_ values of 0.27, 0.42, and 0.21 µM against AsPC-1, MIA PaCa-2, and PANC-1 cell lines, respectively, demonstrated excellent cellular internalization capability and exhibited higher phototoxicity relative to the parent Ce6. The quantitative analyses using Annexin V-PI staining revealed that the **17**-PDT-induced apoptosis was dose-dependent. In pancreatic cell lines, **17** reduced the expression of the anti-apoptotic protein, Bcl-2, and increased the pro-apoptotic protein, cytochrome C, which indicates the activation of intrinsic apoptosis, the primary cause of cancer cell death. Structure–activity relationship studies have shown that the incorporation of additional methyl ester moiety and conjugation to the enone moiety of curcumin enhances cellular uptake and PDT efficacy. Moreover, in vivo PDT testing in melanoma mouse models revealed that **17**-PDT greatly reduced tumor growth. Therefore, **17** might be an effective photosensitizer for PDT anticancer therapy.

## 1. Introduction

Pancreatic cancer remains the most fatal and intractable malignancy and is ranked the 11th most common cancer and 7th leading cause of cancer mortality worldwide [1]. The most prevalent (85% of cases) type of pancreatic cancer is the pancreatic ductal adenocarcinoma (PDAC), which has very poor prognosis with only 9% of the patients surviving for 5 years after diagnosis [2,3]. Gemcitabine is the FDA-approved first-line chemotherapy for PDAC. However, PDAC has shown a lower response to gemcitabine because of primary or acquired resistance and the activation of pro-survival signaling pathways [4,5]. Therefore, the presence of chemotherapy-related side effects and resistance towards first-line treatment necessitates the discovery of more safe and effective drugs for treating pancreatic cancer.

**Figure 1:**
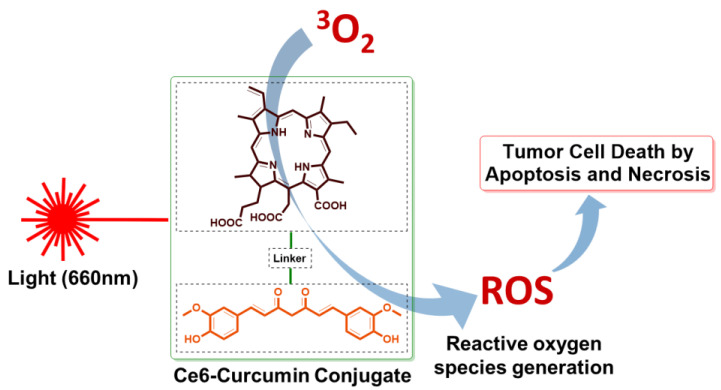
General mechanism of reactive oxygen species generation and PDT-mediated tumor cell death.

Photodynamic therapy (PDT), an emerging cancer treatment option, is the combination of photosensitizer (PS) administration and the irradiation of the harmless visible light of specific wavelengths which induce the cytotoxic reactive oxygen species (ROS) generation by interaction with molecular oxygen at the target site [6,7,8]; the thus formed ROS causes the cancer cell death (Figure 1) either by apoptosis and/or necrosis [9,10]. PDT has numerous advantages as compared to other pre-existing cancer treatment options. It shows relatively low systemic toxicity as the administered PS tends to accumulate more in cancer cells. Similarly, PDT is non-invasive as compared to surgery and it selectively kills tumors because of the ability to irradiate the light to the tumor site only [11,12]. 

Chlorophylls are tetrapyrrolic macrocycles found in plants which, after adequate functionalization, can be efficient photosensitizers due to their remarkable ability to absorb red light (660 nm) and efficiency to generate singlet oxygen [13,14]. In particular, chlorin e6 (Ce6) is a well-known second-generation photosensitizer with high sensitizing efficacy and a rapid elimination time from the body. Researchers have explored the remarkable clinical benefits of Ce6 in the PDT of different types of cancers, including the digestive tract, bladder, melanoma, and nasopharyngeal cancers [15,16,17]. Three different types of carboxylic groups present at the 13-, 15-, and 17-position of Ce6 provide a good platform for medicinal chemists to derivatize it in multiple ways for the discovery of better PS. Several Ce6 derivatives conjugated to peptides, sugars, polyamines, mono-amino acids, and diamine acids were explored by other researchers for their photosensitizing activities [18,19,20,21].

**Figure 2:**
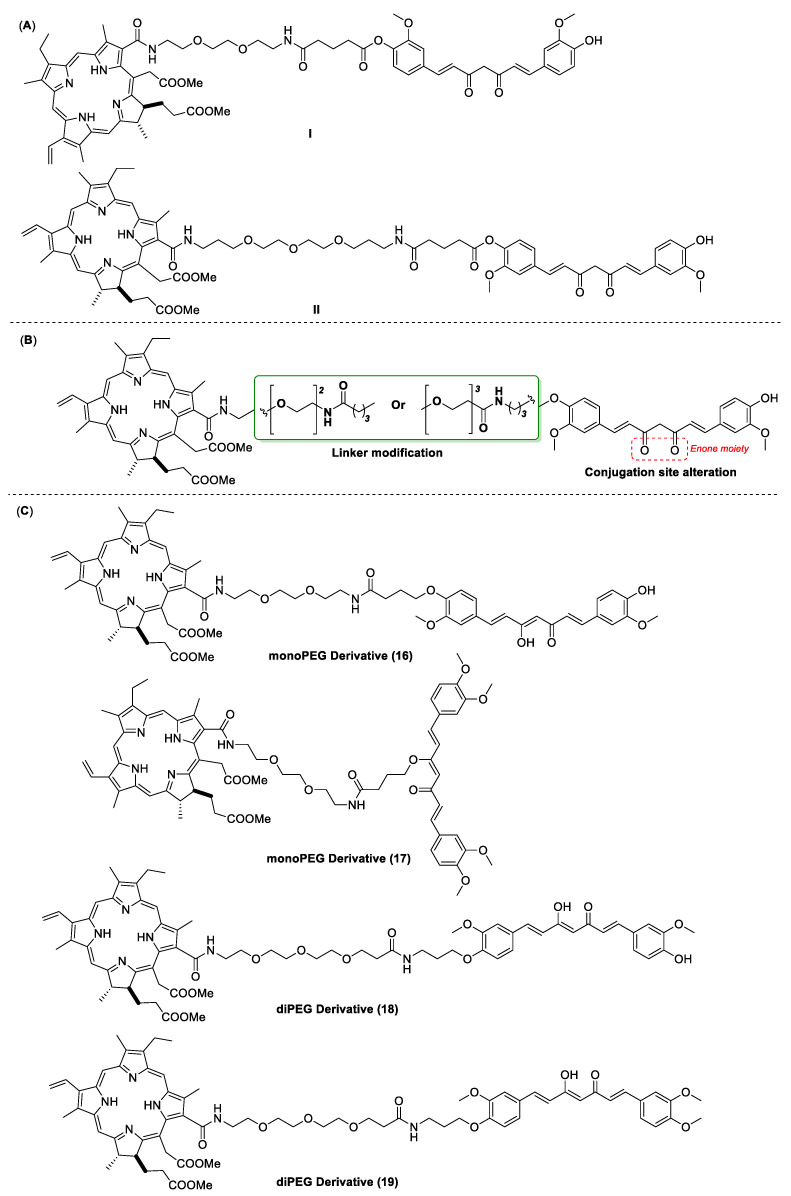
The structure of previously reported Ce6-curcumin derivatives (**A**), strategy for the dug design (**B**), and structure of synthesized compounds in the present study (**C**).

Curcumin, an orange-yellow polyphenolic compound extracted from the rhizome of turmeric plant (*Curcuma longa*) has been extensively used as a spice in Asian cuisine. It has been used in both Ayurveda and traditional Chinese medicine to treat skin diseases, infection, injuries, and digestive disorders [22,23]. Curcumin and its derivatives have been extensively studied in the past two decades because of their promising biological effects, such as anticancer, anti-inflammatory, and antioxidant activities [24]. Over 100 cellular targets of curcumin have been identified to date, including enzymes, cytokines, receptors, and transcription factors [25,26]. Curcumin exerts its anticancer activity by inducing apoptosis and suppressing different types of cell-signaling pathways, leading to the inhibition of proliferation and tumor invasion [27,28]. Several researchers have demonstrated the anticancer activity of curcumin against lung cancer, breast cancer, prostate cancer, head and neck squamous cell carcinoma, brain cancer, and pancreatic cancer [29]. Recently, curcumin has been used in combination with 5-Fluorouracil, Gemcitabine, Cisplatin, and Doxorubicin as a chemotherapy sensitizing agent [30,31]. In the quest for developing novel PS, we have previously reported Ce6-curcumin derivatives with different types of linker such as hexane, propane, monoPEG, and diPEG. Among them, PEG containing derivatives **I** and **II** (Figure 2A) displayed significant PDT efficacy against pancreatic cancer cell lines [32]. To further investigate the effect of linker modification and conjugation site alteration, we rationally designed and synthesized novel Ce6-curcumin derivatives (Figure 2B) and assessed their PDT efficacy against pancreatic carcinoma in vitro and murine melanoma model in vivo.

## 2. Results and Discussion

### 2.1. Synthetic Chemistry

**Scheme 1:**
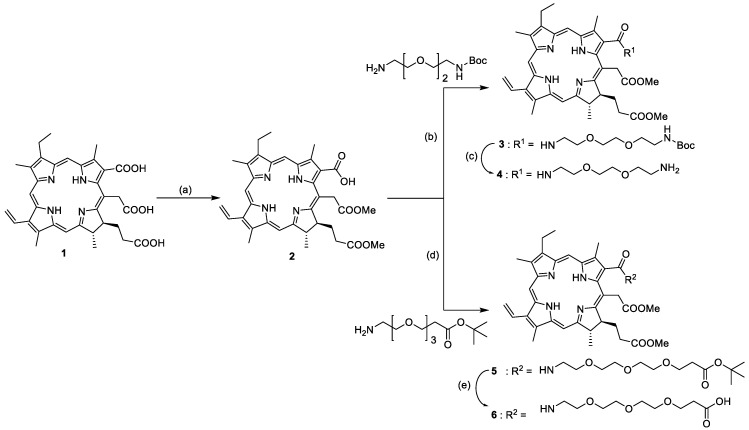
The general synthetic method of DME Ce6-monoPEG-NH_2_ (**4**) and DME Ce6-diPEG-COOH (**6**). Reagent and conditions: (a) 5% H_2_SO_4_, MeOH, rt, 12 h, 88% yield; (b) EDCI, HOBt, DIPEA, CH_2_Cl_2_, rt, 12 h, 63% yield; (c) TFA, CH_2_Cl_2_, rt, 12 h, 87% yield; (d) HATU, HOBt, DIPEA, CH_2_Cl_2_, rt, 24 h, 48% yield; (e) 15% TFA, CH_2_Cl_2_, rt, 30 min.

**Scheme 2:**
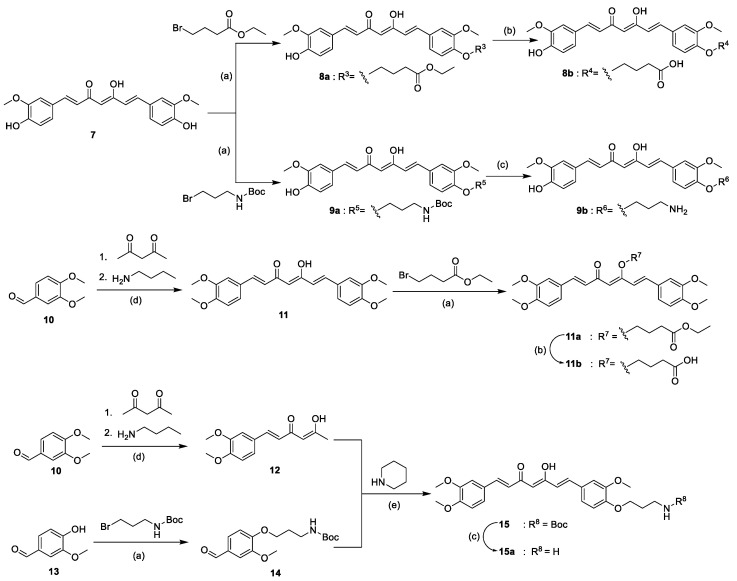
The general synthetic method of curcumin derivatives (**8b**, **9b**, **11b**, and **15a**). Reagent and conditions: (a) K_2_CO_3_, DMF, 80 °C, 12 h, 19–77% yield; (b) LiOH, THF/H_2_O, rt, 12 h, 30% yield; (c) 15% TFA, CH_2_Cl_2_, rt, 30 min, 83% yield; (d) B_2_O_3_, (*n*-BuO)_3_B, EtOAc, 80 °C, 2.5 h, 36–52% yield; (e) B_2_O_3_, (*n*-BuO)_3_B, HCl, EtOAc, 85 °C, 2 h, 11% yield.

**Scheme 3:**
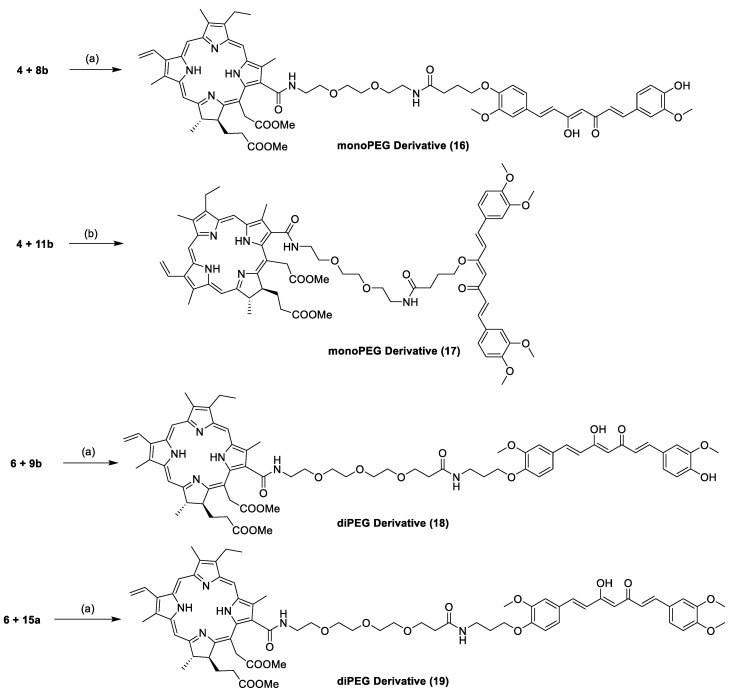
The general synthetic method of final compounds **16**, **17**, **18**, and **19**. Reagent and conditions: (a) HBTU, HOBt, DIPEA, CH_2_Cl_2_, rt, 12 h, 26–37% yield; (b) EDCI, HOBt, DIPEA, CH_2_Cl_2_, rt, 12 h, 38% yield.

The synthetic method of the Ce6-curcumin derivatives is illustrated in Scheme 1, Scheme 2 and Scheme 3. 2,2′-(Ethane-1,2-diylbis(oxy))bis(ethan-1-amine) (monoPEG) and 3-[2-[2-(2-aminoethoxy)ethoxy]ethoxy]propanoic acid (diPEG) linkers were used for conjugating Ce6 with curcumin. The dimethyl ester of Ce6 (**2**) (DME Ce6) was obtained by the selective esterification of Ce6 (**1**) using 5% of H_2_SO_4_ in methanol [32]. The DME Ce6 (**2**) was activated using 1-hydroxybenzotriazole (HOBt) and 1-ethyl-3-(3-(dimethylamino)propyl)-carbodiimide hydrochloride (EDCI) and coupled individually with commercially available *tert*-butyl (2-(2-(2-aminoethoxy)ethoxy)ethyl)carbamate and *tert*-butyl 3-(2-(2-(2-aminoethoxy)ethoxy)ethoxy)propanoate to give *tert*-butyl-protected compounds **3** and **5**, which were deprotected with trifluoroacetic acid (TFA) to afford Ce6-monoPEG-NH_2_ (**4**) and Ce6-diPEG-COOH (**6**) intermediates, respectively (Scheme 1). The selective S_N_2 reaction of curcumin-enol analogue (**7**) with ethyl 4-bromobutanoate or *tert*-butyl (3-bromopropyl)carbamate individually yielded **8a** and **9a**. The two compounds underwent lithium hydroxide (LiOH)/TFA-mediated deprotection to yield curcumin intermediates **8b** and **9b**, respectively. The tetramethoxy curcumin derivative (**11**) was synthesized by condensing acetyl acetone with aryl aldehyde (**10**) in the presence of n-butyl amine. The resulting tetramethoxy curcumin (**11**) was O-alkylated with ethyl 4-bromobutanoate to afford intermediate (**11a**) followed by ester hydrolysis in the presence of LiOH to obtain the tetramethoxy curcumin monocarboxylic acid derivative (**11b**). **15a** was obtained as a result of the condensation reaction between **12** and **14** followed by TFA-mediated Boc deprotection. **12** was obtained from the treatment of **10** by excess of pentanedione to avoid aldol condensation taking place at both terminal ends. **14** was prepared by the alkylation of aryl aldehyde **13** (Scheme 2). Finally, the HOBt/EDCI coupling of **8b** or **11b** with DME Ce6-monoPEG-NH_2_ (**4**) afforded Ce6-curcumin derivatives **16** and **17**. Compounds **18** and **19** were synthesized via an amide coupling reaction between **9b** or **15a** with DME Ce6-diPEG-COOH (**6**) (Scheme 3). The synthesized compounds were characterized using NMR, ESI-MS, and UV–visible spectroscopy. Ce6-curcumin derivatives possessed characteristic aromatic proton peaks of curcumin moiety around 7 ppm. The proton and carbon peaks, corresponding to the PEG linker used (-O-CH_2_-CH_2_-O-), were observed at 3–4 ppm and 60–70 ppm, respectively. Similarly, characteristic downfield-shifted enol and the keto carbon of curcumin was observed at 180–190 ppm. The ^1^H & ^13^C NMR, ESI-MS spectra of selected compounds are included in the Supplementary Information (SI Sections S1 and S2).

### 2.2. Analysis of Photophysical Properties and Singlet Oxygen Generation Capability 

**Figure 3:**
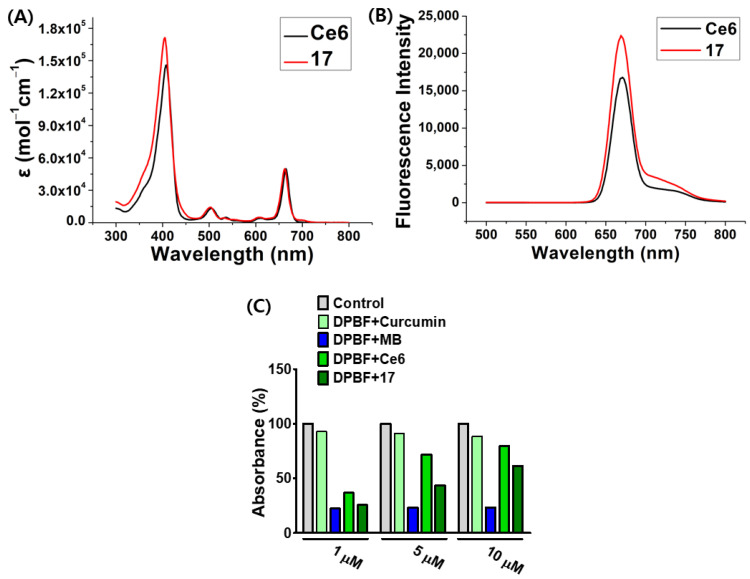
The photophysical properties of Ce6-curcumin derivative: (**A**) absorption spectra; (**B**) fluorescence spectra; and (**C**) the singlet oxygen generation of Ce6-curcumin derivative (**17**) in three different concentrations (1, 5, and 10 µM), using DPBF (50 µM) as the control.

The UV–vis absorption and fluorescence of representative compound **17** in DMSO were analyzed and compared with starting material Ce6. The absorption spectra of all compounds were typical to Ce6 derivatives with one Soret band and Q-bands. Both Ce6 and Ce6-curcumin conjugate **17** effectively absorbed the red light with the major Soret peak at λ_max_ = 405–408 nm (Figure 3A). The Soret band of Ce6 is sharp, while Ce6-curcumin derivatives have a broad peak due to curcumin conjugation. Similarly, the tested compounds showed fluorescence at 650–700 nm (Figure 3B). **17** was found to have higher absorbance and fluorescence properties as compared to Ce6 at the same concentration of 20 µM.

The primary process in the photodynamic destruction of cancer cells is singlet oxygen production. Using 1, 3-diphenylisobenzofuran (DPBF) as a singlet oxygen trap, the capacity of Ce6-curcumin derivative and Ce6 to create singlet oxygen (^1^O_2)_ under photosensitizing conditions was investigated in DMSO. The irreversible reaction between DPBF and ^1^O_2_ can be observed by monitoring the drop in DPBF absorption intensity at 418 nm. A solution of the selected most active Ce6-curcumin derivative (**17**), Ce6, methylene blue (MB), and DPBF quenched the DPBF absorption band at 418 nm when exposed to a 660 nm laser (Figure 3C). DPBF and curcumin show absorption maxima in the same region (400–450 nm), making it more difficult to analyze the results due to the overlap of the two bands. Therefore, the absorption of curcumin only has been subtracted from the obtained result. Curcumin-only did not display significant singlet generation (88.65–92.73%). MB displayed the most significant ^1^O_2_ generation (22.22%), which is comparable to Ce6 (36.63%) and **17** (25.51%) at 1 µM concentration. Interestingly, Ce6-curcumin derivative **17** generated 11.12% higher singlet oxygen at 1 µM concentration capability as compared to Ce6 alone. In contrast, Ce6 and **17** displayed lower singlet oxygen generation at 5 and 10 µM, while the singlet oxygen generation potency of MB remained similar at all three tested concentrations. 

### 2.3. Cellular Uptake Assay

**Figure 4:**
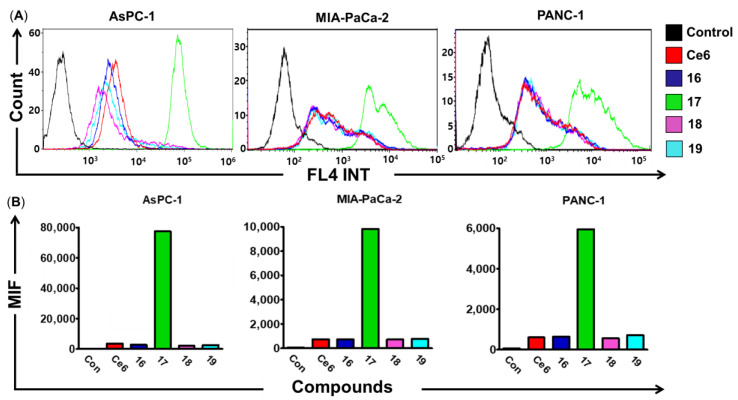
Flow cytometric analysis after the treatment with 5 μΜ of Ce6, **16**, **17**, **18** and **19**, respectively, in the AsPC-1, MIA PaCa-2, and PANC-1 cell lines for 3 h. (**A**) The cellular uptake study in the abovementioned cells; (**B**) the mean fluorescence intensity (MFI) generated against the tested cell lines.

The ability of PS to cross the cell membrane is vital for better PDT. A higher cellular uptake of PS results in enhanced PDT efficacy. According to flow cytometry, pretreatment with **17** (5 µM) for 3 h boosted cellular uptake in AsPC-1, MIA-PaCa-2, and PANC-1 cells, whereas the uptake of **16**, **18**, **19**, and Ce6 (5 µM) was less (Figure 4A). **16**, **18**, **19**, and Ce6 showed similar cellular uptake in all the tested cell lines. However, the fluorescence intensity of **17** was 22.2-, 9.8-, and 13-fold higher (Figure 4B) than free Ce6 after 3 h in AsPC-1, MIA-PaCa-2, and PANC-1 cells, respectively, which corroborates with our prior study [32]. Several types of Ce6 derivatives, such as PEGylated, have been reported to exhibit enhanced cellular uptake [33,34,35]. This fact allows for us to conclude that **17** penetrates well into the abovementioned cell lines.

### 2.4. Cytotoxicity of Ce6-Curcumin Derivatives In Vitro 

**Figure 5:**
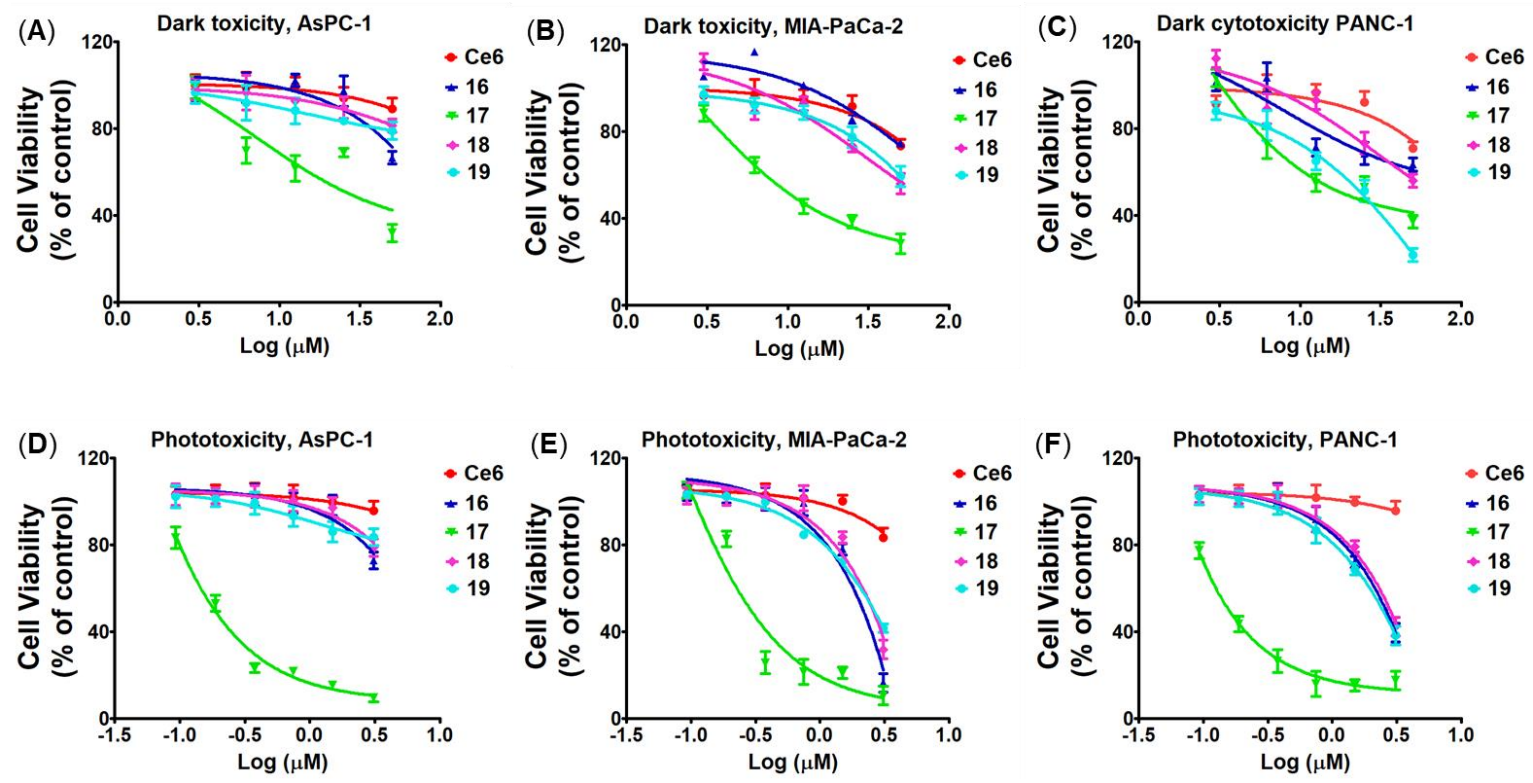
The dark toxicity of Ce6-curcumin derivatives **16**, **17**, **18**, and **19** against (**A**) AsPC-1 cells; (**B**) MIA PaCa-2 cells; (**C**) PANC-1 cells and phototoxicity against (**D**) AsPC-1 cells; (**E**) MIA PaCa-2 cells; (**F**) PANC-1 cells using Ce6 as a control. The data points are represented as the mean ± SD (*n* = 3) of three separate experiments.

The dark cytotoxicity of Ce6 and its synthesized Ce6-curcumin derivatives **16**, **17**, **18**, **19** were evaluated in AsPC-1, MIA PaCa-2, and PANC-1 cell lines (Figure 5). The cell viability of Ce6 and its synthesized derivatives was determined by a standard MTT assay to evaluate the cell viability after incubation for 72 h. It is worth noting that most of the compounds in the concentration of 50 μM were not toxic and displayed dark toxicity with IC_50_ ≥ 50 µM against the tested cell lines as compared to that of the control. However, **17** against all tested cell lines and **19** against PANC-1 cells exhibited higher dark cytotoxicity. Their IC_50_ values can be referred to in Figure 5A–C and Table 1. The enhanced dark cytotoxicity of Ce6-curcumin derivative in contrast to the parent Ce6 molecule might have been contributed by curcumin, a well-known anticancer agent. Moreover, it was determined that the tested compound resulted in a rapid increase in photo-induced cytotoxicity in the concentration range of 0.09–3.1 μM when the light (660 nm, 50 mW, 5 J/cm^2^) was irradiated after drug treatment. Photo-induced cytotoxicity of **17** on the cell lines AsPC-1, MIA PaCa-2, and PANC-1 was higher than that of Ce6 (Figure 5D–F). Besides that, **16**, **18**, and **19** showed similar phototoxicity as that of Ce6 (IC_50_ ≥ 3.1 µM) (Table 1). The enhanced cytotoxicity of **17** can be linked to its higher cellular uptake, evident by the FACS analysis and the synergistic interaction of Ce6 and curcumin [36,37,38]. It has been shown that the combination of curcumin with 5-ALA significantly inhibited the viability of human colorectal adenocarcinoma cells (Caco-2) by 62.4% as compared to curcumin and 5-ALA alone [39].

### 2.5. Compound **17** Mediates the Pancreatic Cancer Cell Death by Intrinsic Apoptosis

**Figure 6:**
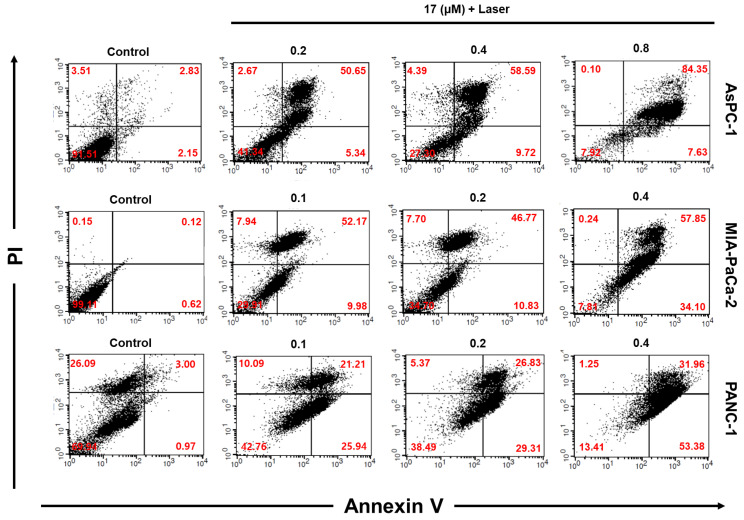
Flow cytometry analysis of AsPC-1, MIA-PaCa-2, and PANC-1 cells with Annexin V-FITC/PI double staining depicting apoptosis after PDT: cells were pretreated with or without the different concentration of **17** for 3 h followed by irradiation with a light dose of 5 J/cm^2^ prior to analysis. Cells undergoing early and late apoptosis can be seen in the lower right and upper right quadrants. The images shown are representative of one independent experiment.

We selected **17** for additional in vivo and in vitro studies because it exhibited the best cellular uptake and had better cytotoxicity against pancreatic cancer cell lines. Using an Annexin V-PI kit for flow cytometry, the apoptotic profiles of **17** followed by PDT on AsPC-1, MIA PaCa-2, and PANC-1 cells were assessed. The four-quadrant in Figure 5 shows the flow cytometry analysis of live, early-stage apoptosis, late-stage apoptosis, and dead cells for **17** in the above-mentioned cell lines. We found that the treatment of AsPC-1 with **17** (0.2–0.8 µM) followed by PDT for 72 h increased the number of late apoptotic cells in a dose-dependent manner (Figure 6 and Table 2). At a concentration of 0.8 µM of **17** along with PDT, the highest percentage of apoptotic AsPC-1 cells (91.98%) was observed. However, treatment of MIA PaCa-2 and PANC-1 cells with **17**-PDT (0.1–0.4 µM) increased the number of both late and early apoptotic cells in a dose-dependent manner. Moreover, the sum of both the late and early apoptotic cell population was the highest (91.95% and 85.34%) in MIA-PaCa-2 and PANC-1 cells, respectively, at a concentration of 0.4 µM. In the light of these findings, **17** inhibits the pancreatic cancer cells by inducing apoptosis. Similar results of various Ce6 derivatives associated with PDT have been reported. Gao et al. reported that the Ce6-guanidine derivative associated with PDT induced apoptotic cell death in A549, the lung cancer cell line [40]. Similarly, Maltotriose-Ce6 derivatives have been shown to have both apoptotic and necrotic pathways to kill the tested breast cancer cell line (EMT6) [41]. 

**Figure 7:**
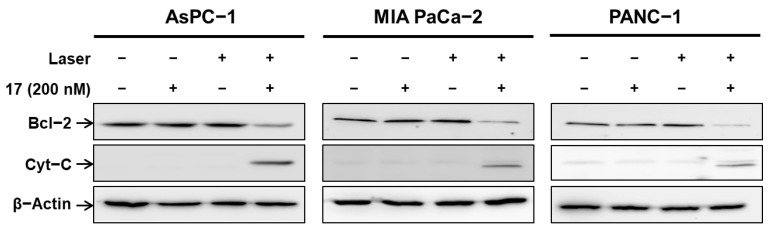
The effect of **17**-PDT on the protein expression pattern of anti-apoptotic and pro-apoptotic protein markers in AsPC-1, MIA-PaCa-2, and PANC-1 cells. The total proteins were extracted and separated by SDS-PAGE. The proteins were subjected to immunoblotting using anti-Bcl-2 and anti-cytochrome C antibody. The images shown are representative of one independent experiment.

The Bcl-2 family proteins play a crucial role in the process of cell death by apoptosis. Bcl-2 binds to the Bax protein and causes mitochondrial outer membrane permeabilization, leading to apoptosis prevention [42]. Similarly, the cytochrome C protein is related to an inner mitochondrial membrane which enters into cytosol, activating caspase-9 after permeabilization of the outer mitochondrial membrane by Bcl-2 [43]. To further understand the mechanism of apoptosis, Western blot analysis was used to assess the expression of apoptosis-related proteins (Bcl-2 and cytochrome C) after treatment with **17** followed by PDT. We observed that cells treated with **17**-PDT at the dose of 0.2 µM showed decreased levels of the anti-apoptotic protein, Bcl-2, compared with untreated cells. Likewise, regarding the pro-apoptotic factor, cytochrome C levels were increased with **17**-PDT compared to the untreated groups. Interestingly, groups who received simply laser or the **17** treatment showed no differences in the expression of the Bcl-2 or cytochrome C proteins (Figure 7). This result is in line with our previous finding, where the most active Ce6-curcumin derivative displayed a similar pattern of apoptotic protein expression against the AsPC-1 cell line [32]. Studies on the PDT efficacy of another Ce6 derivative (YLG-1) by Shen et al. have revealed to generate 44.03% and 29.0% of the apoptotic population against the SW1990 and PANC-1 cell lines, respectively, which was further supported by the overexpression of BAX and under-expression of Bcl-2 proteins [44]. Therefore, this result indicated that **17**-PDT triggered apoptosis-related pancreatic cancer cell death.

### 2.6. Compound **17**-PDT Efficacy on Xenograft Mouse Model Using B16F10 Melanoma Cell

**Figure 8:**
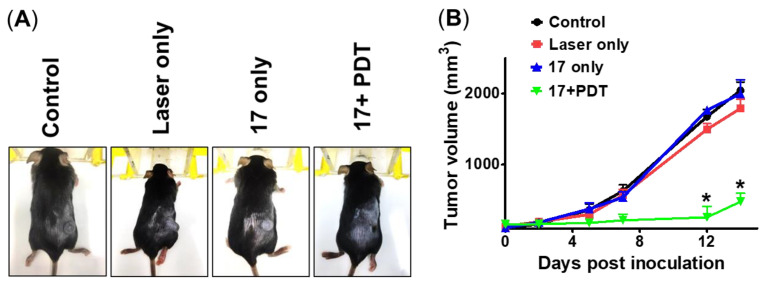
The growth inhibitory effect of **17**-PDT on tumor-bearing mice. (**A**) B16F10 (1 × 10^5^ cells/mL) were subcutaneously injected into the right flank of C57BL/6 mice. The intravenous administration of **17** (2.5 mg/kg) was carried out for 3 h, followed by the irradiation of a 660 nm laser diode, (LD, LEMT, Belarus) at a dose of 100 J/cm^2^ in the fourth group, while only laser and **17** was given to second and third groups. (**B**) The tumor volumes of the 4 groups are plotted from day 0 to day 14 after the inoculation. The data are presented as mean ± SD, *n* = 4. * p  <  0.05 compared to the control tumor.

In order to validate the in vitro efficacy of **17**-PDT, we further investigated its effectiveness in a mice xenograft model. We have examined the PDT efficacy of **17** in vivo utilizing B16F10, a murine melanoma cancer cell. Mice were intravenously injected with **17** (2.5 mg/kg) followed by laser irradiation (3 h post-injection) when the tumor volume reached 100 mm^3^ (Figure 8A). We observed a significant inhibition of tumor growth in mice treated with the dose of 2.5 mg/kg of compound followed by PDT. However, when compared to the control (Ce6)-treated group, the laser- and **17**-alone-treated groups displayed a similar tumor growth pattern (Figure 8B). A previous study by Kim et al. also suggested that the hyaluronic acid–Ce6-mediated PDT significantly inhibited tumor growth in the melanoma mouse model as compared to free Ce6 [45]. Similarly, Ce6-GFLG conjugates, as immune checkpoint inhibitors, have been shown to lower pulmonary metastatic colorectal cancer (CT26) growth in mouse models [46]. To the best of our knowledge, we report for the first time that **17**-PDT effectively suppresses cancer growth by the apoptotic pathway. 

### 2.7. Effect of **17** in Mice Behavior, Growth, and Its Pharmacokinetics

**Figure 9:**
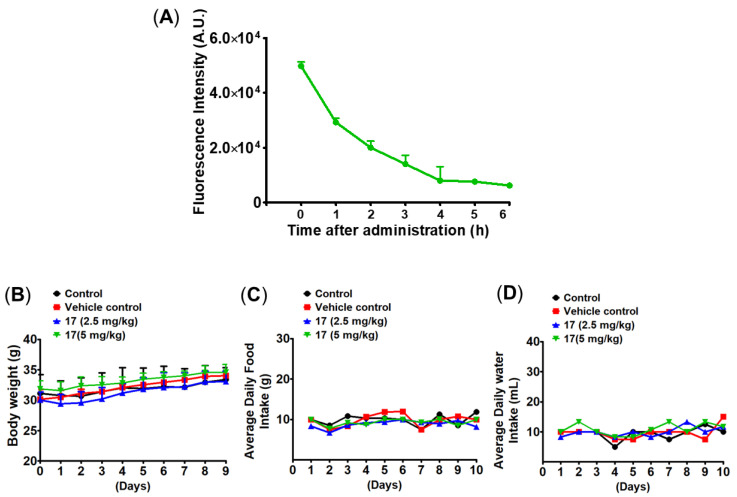
In vivo mice toxicity and pharmacokinetics evaluation of **17**. (**A**) Time-dependent fluorescence intensity changes in plasma following the intravenous injection of 2.5 mg/kg of **17**. Mice behavior and weight changes after receiving the intravenous administration of **17** (2.5 and 5 mg/kg) for 10 days as compared to the vehicle control and control group. No significant differences in the (**B**) body weights, (**C**) daily food consumption, and (**D**) water intake were noted in **17**-treated animals over the course of 10 days as compared to the control groups. The data are represented as the mean ± SD for each group (*n* = 3).

As a result of the overall findings and drug-like profile, **17** was chosen as our lead for in vivo toxicity and pharmacokinetics investigations as a preliminary characterization in mouse. We have studied the pharmacokinetics of **17** following intravenous injection and analyzed the concentration of **17** in plasma. The distribution of **17** in mouse plasma at times ranging from 0 to 6 h after the intravenous injection of 2.5 mg/kg was estimated by the fluorescence intensity. The distribution of **17** in the plasma following drug delivery is depicted in Figure 9A. The fluorescence intensity of **17** in the plasma peaked at 1 h after injection and exponentially decreased continuously until it reached a low level at 4 h. Six hours after injection, the fluorescence of **17** was slowed down.

Body weight and behavioral patterns of the mice were also observed throughout the experiment to determine the toxicity of **17**. Mice were intravenously injected with or without the vehicle (10% DMSO, 10% Tween 80, and 80% water), low doses (2.5 mg/kg), and high doses (5 mg/kg) of Ce6, respectively. Without any significant difference, the growth of all mouse groups increased in the same way (Figure 9B). All groups of mice showed a similar daily intake of food and water (Figure 9C,D). Following the administration of **17** in the high and low doses (SI Section S4), no deaths, convulsions, aggression, aberrant behaviors, etc., were noted in the mice. Additionally, there was no hair loss and the breathing pattern matched that of the control group. All these parameters are visible in accordance with the lack of toxicity due to **17**.

### 2.8. Structure—Activity Relationship (SAR) Study and Molecular Docking Analysis 

**Figure 10:**
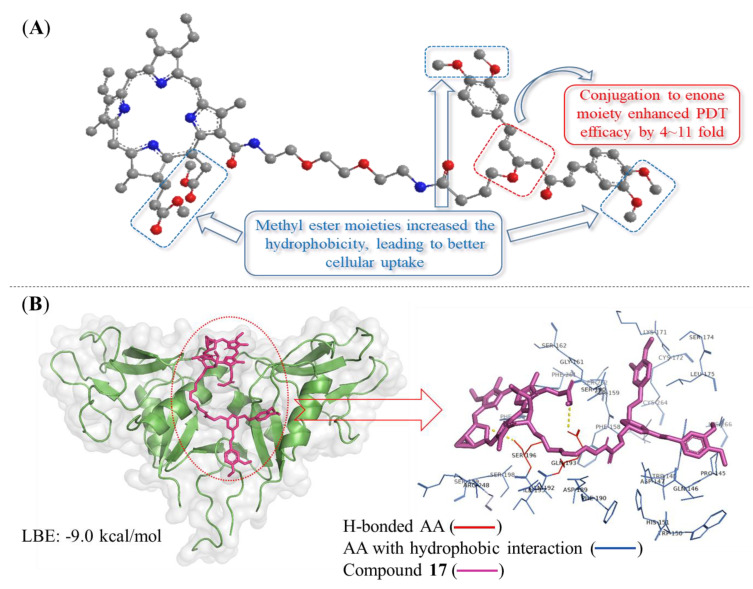
(**A**) A structure–activity relationship study of the synthesized compounds. (**B**) A predicted binding mode of **17** to the LDL protein (PDB code: 1YPQ). The lowest binding energy (LBE) and amino acid (AA) residues involved are given (H-bonds are represented as the yellow dotted line).

Linker modification and conjugation site alteration to enone moiety allowed for us to conduct the SAR study relative to previously reported compounds **I** and **II** (Figure 10A). Incorporation of methyl ester moieties on the 15- and 17-carboxylic acid groups of Ce6 and feruloyl moieties of curcumin increased the hydrophobicity, which in turn might have enhanced the affinity for the cell membrane to increase the cellular uptake of **17** [47]. Previously reported monoPEG derivative (**I**) containing two carbonyl groups in the linker displayed better phototoxicity (IC_50_: 0.035–0.04 µM) as compared to the monoPEG derivative **16** (IC_50_: ≥2.05), which had one carbonyl group in the linker. Interestingly, Ce6-monoPEG linked to the enone moiety of curcumin (**17**) significantly enhanced PDT efficacy by ≥11.48-, 4.88-, and 10.23-fold against AsPC-1, MIA PaCa-2, and PANC-1 cell lines, respectively. On the other hand, the diPEG derivative (**18** and **19**) displayed comparatively better efficacy than the previously reported diPEG derivative (**II**) conjugated with two carbonyl containing linkers. Moreover, lipoproteins, particularly low-density lipoproteins (LDL), play an important role in the transport of PS to the tumor site, governing its efficacy and selectivity [48]. Therefore, we have decided to analyze the binding affinity of synthesized compounds with the LDL. Among them, **17** was bound to the largest cavity (1042 Å^3^) of the protein with lowest binding energy of −9.0 kcal/mol. **17** interacted with the SER 196 and GLN 193 residues of the LDL protein via hydrogen bonding (Figure 10B). The binding pose of other derivatives—**16**, **18**, and **19**—were included in Supplementary Information (SI Section S4). This finding supports the better cellular uptake and PDT efficacy of **17**.

## 3. Materials and Methods

### 3.1. General Information

Commercially available starting materials and reagents were purchased from Sigma-Aldrich, TCI Chemicals and Alfa-Aesar, and used without further purification. Column chromatography was carried out using silica gel (230–400 mesh, Zeochem, Lake Zurich, Switzerland). Thin layer chromatography (TLC) was performed on silica gel plates (Kieselgel 60 F_254_, Merck, Darmstadt, Germany) with a layer thickness of 0.25 mm. Medium pressure liquid chromatography (MPLC) purifications were performed by CombiFlash RF+ UV (Redisep@from Teledyne Isco, Lincoln, NE, USA). ^1^H NMR (400 MHz) and ^13^C NMR (100 MHz) spectra were recorded in Bruker 400 spectrometer using tetramethylsilane (TMS) as the internal standard. All the chemical shifts values (*δ*) were recorded in parts per million (ppm) and coupling constants (*J*) in hertz (Hz). ESI mass spectrometric data were obtained on the Shimadzu LC-MS system. Molecular docking analysis was performed using a web-based server for the protein–ligand docking program (CB-Dock) [49].

### 3.2. Chemistry

The dimethyl esters of Ce6 (**2**), Ce6-MonoPEG-NHBoc (**3**), and Ce6-MonoPEG-NH_2_ (**4**) were synthesized and characterized using a previously reported method [32].

#### 3.2.1. Synthesis of Ce6-DiPEG-COOtBu (**5**)

To the solution of compound **2** (500 mg, 0.8 mmol) dissolved in CH_2_Cl_2_ (10 mL), HBTU, O-benzotriazol-1-yl-tetramethyluronium hexafluorophosphate (455 mg, 1.2 mmol), and N,N-diisopropylethylamine (0.28 mL, 1.6 mmol) were added. Then, this mixture was stirred at room temperature for 20 min. *Tert*-butyl 3-(2-(2-(2-aminoethoxy)ethoxy)ethoxy)propanoate (333 mg, 1.2 mmol) was added to the reaction mixture and stirred at room temperature for 24 h. The resulting solution was extracted with ethyl acetate and water. The combined organic phase was dried with magnesium sulfate and filtered. After that, solvent was concentrated in vacuo. The crude mixture was purified by MPLC (0~10% MeOH: 100~90% CH_2_Cl_2_) to afford the desired product (340 mg, 48%). ^1^H NMR (400 MHz, CDCl_3_) *δ* 9.70 (s, 1H), 9.64 (s, 1H), 8.81 (s, 1H), 8.09 (dd, *J* = 17.8, 11.6 Hz, 1H), 7.00–6.81 (m, 1H), 6.36 (dd, *J* = 17.8, 1.3 Hz, 1H), 6.15 (dd, *J* = 11.5, 1.3 Hz, 1H), 5.53 (d, *J* = 18.8 Hz, 1H), 5.28 (d, *J* = 18.8 Hz, 1H), 4.47 (dd, *J* = 14.5, 7.2 Hz, 1H), 4.39 (d, *J* = 7.7 Hz, 1H), 4.13–4.03 (m, 1H), 3.91–3.85 (m, 3H), 3.79 (dt, *J* = 18.2, 9.1 Hz, 5H), 3.72 (s, 5H), 3.59 (t, *J* = 15.2 Hz, 8H), 3.49 (s, 4H), 3.42 (s, 4H), 3.32 (s, 3H), 2.54 (dt, *J* = 15.7, 7.9 Hz, 1H), 2.36–2.18 (m, 3H), 2.17–2.00 (m, 1H), 1.89–1.75 (m, 1H), 1.71 (dd, *J* = 9.5, 7.4 Hz, 6H), 1.30 (s, 9H); ESI-MS, *m/z*: 885.30 [M+H]^+^.

#### 3.2.2. Synthesis of Ce6-DiPEG-COOH (**6**)

To the solution of compound **5** (200 mg, 0.24 mmol) dissolved in CH_2_Cl_2_ (5 mL), 15% TFA in CH_2_Cl_2_ (3 mL) was added and stirred at room temperature for 30 min. The resulting solution was extracted with H_2_O. After that, the organic solvent was concentrated in vacuo. The resulting residue was used in next step without further purification. ^1^H NMR (400 MHz, MeOD) *δ* 9.65 (s, 1H), 9.48 (s, 1H), 8.90 (s, 1H), 8.01–7.89 (m, 1H), 6.20 (d, *J* = 15.6 Hz, 1H), 6.01–5.98 (m, 1H), 5.50 (d, *J* = 19.1 Hz, 1H), 5.28 (d, *J* = 19.2 Hz, 1H), 4.60–4.39 (m, 3H), 4.37–4.32 (m, 1H), 3.96–3.79 (m, 3H), 3.71–3.59 (m, 10H), 3.55 (s, 3H), 3.47 (s, 5H), 3.42–3.29 (m, 7H), 3.15–3.12 (m, 3H), 2.68–2.55 (m, 1H), 2.34–2.10 (m, 4H), 1.71–1.59 (m, 7H); ESI-MS, *m/z*: 828.10 [M+H]^+^.

#### 3.2.3. Synthesis of Ethyl 4-(4-((1E,3Z,6E)-3-hydroxy-7-(4-hydroxy-3-methoxyphenyl)-5-oxohepta-1,3,6-trien-1-yl)-2-methoxyphenoxy)butanoate (**8a**)

To the mixture of compound **7** (500 mg, 1.357 mmol), ethyl 4-bromobutanoate (0.194 mL, 1.357 mmol) in DMF (5 mL) and potassium carbonate (188 mg, 1.357 mmol) were added at 0 °C. Then, the reaction mixture was stirred at room temperature for 12 h. After the removal of the solvent in vacuo, the residue was dissolved in ethyl acetate. Then, this was filtered to an insoluble solid, washed sequentially with water, with 5% NaHCO_3_ in water, and saturated NaCl in water. The organic phase was dried with anhydrous Na_2_SO_4_. The crude mixture was purified by MPLC (0~10% MeOH: 100~90% CH_2_Cl_2_) (120 mg, 19%) to afford the desired product. ^1^H NMR (400 MHz, CDCl_3_) *δ* 7.63–7.56 (m, 2H), 7.12 (dt, *J* = 8.3, 2.0 Hz, 2H), 7.06 (dd, *J* = 8.7, 2.0 Hz, 2H), 6.94 (d, *J* = 8.2 Hz, 1H), 6.89 (d, *J* = 8.3 Hz, 1H), 6.51 (d, *J* = 3.4 Hz, 1H), 6.47 (d, *J* = 3.5 Hz, 1H), 5.82 (s, 1H), 4.20–4.09 (m, 4H), 3.95 (s, 3H), 3.91 (s, 3H), 2.54 (t, *J* = 7.2 Hz, 2H), 2.18 (p, *J* = 6.8 Hz, 2H), 1.29–1.23 (m, 5H); ESI-MS, *m/z*: 483.10 [M+H]^+^.

#### 3.2.4. Synthesis of 4-(4-((1E,3Z,6E)-3-Hydroxy-7-(4-hydroxy-3-methoxyphenyl)-5-oxohepta-1,3,6-trien-1-yl)-2-methoxyphenoxy)butanoic Acid (**8b**)

Compound **8a** (100 mg, 0.207 mmol) and lithium hydroxide monohydrate (17.39 mg, 0.414 mmol) were dissolved in THF (2 mL) and water (2 mL) and stirred for 30 min at room temperature. The residue was acidified with HCl and extracted with CH_2_Cl_2_. After that, organic solvent was concentrated in vacuo. The resulting residue was used in the next step without further purification. ^1^H NMR (400 MHz, CDCl_3_) *δ* 7.58 (d, *J* = 15.8 Hz, 2H), 7.21–6.97 (m, 4H), 6.90 (dd, *J* = 8.2, 2.9 Hz, 2H), 6.49 (dd, *J* = 15.8, 5.7 Hz, 2H), 5.31 (s, 1H), 4.11 (t, *J* = 6.3 Hz, 2H), 3.94 (s, 3H), 3.90 (d, *J* = 6.6 Hz, 3H), 2.55 (t, *J* = 7.2 Hz, 2H), 2.25–2.07 (m, 2H); ESI-MS, *m/z*: 455.00 [M+H]^+^.

#### 3.2.5. Synthesis of *Tert*-butyl (3-(4-((1E,3Z,6E)-3-hydroxy-7-(4-hydroxy-3-methoxyphenyl)-5-oxohepta-1,3,6-trien-1-yl)-2-methoxyphenoxy)propyl)carbamate (**9a**)

Compound **7** (500 mg, 1.357 mmol), *tert*-butyl (3-bromopropyl)carbamate (549 mg, 2.307 mmol), and potassium carbonate (244 mg, 1.764 mmol) were dissolved in DMF (10 mL) and stirred at room temperature for 12 h. The resulting solution was extracted with ethyl acetate and water. The combined organic phase was dried with magnesium sulfate and filtered. After that, solvent was concentrated in vacuo. The crude mixture was purified by MPLC (20~80% EA: 80~20% Hex) to afford the desired product (194 mg, 27%). ^1^H NMR (400 MHz, CDCl_3_) *δ* 7.60 (dd, *J* = 15.8, 1.7 Hz, 2H), 7.21–7.02 (m, 4H), 6.94 (d, *J* = 8.2 Hz, 2H), 6.86 (d, *J* = 8.3 Hz, 2H), 6.49 (dd, *J* = 15.8, 5.7 Hz, 2H), 5.85–5.78 (m, 2H), 4.12 (dt, *J* = 10.7, 6.4 Hz, 2H), 3.94 (d, *J* = 8.0 Hz, 12H), 3.38 (d, *J* = 5.5 Hz, 2H), 2.10–1.76 (m, 2H), 1.45 (d, *J* = 7.5 Hz, 9H); ESI-MS, *m/z*: 526.00 [M+H]^+^.

#### 3.2.6. Synthesis of (1E,4Z,6E)-7-(4-(3-Aminopropoxy)-3-methoxyphenyl)-5-hydroxy-1-(4-hydroxy-3-meth-oxyphenyl)hepta-1,4,6-trien-3-one (**9b**)

To the solution of compound **9a** (136 mg, 0.38 mmol) dissolved in MC (10 mL) was added 15% TFA in MC (5 mL) and stirred at room temperature for 30 min. The resulting solution was extracted with H_2_O. After that, organic solvent was concentrated in vacuo. Crude mixture was purified by MPLC (0~30% MeOH: 100~70% CH_2_Cl_2_) to afford the desired product (91 mg, 83%). ESI-MS, *m/z*: 426.00 [M+H]^+^.

#### 3.2.7. Synthesis of (1E,6E)-1,7-Bis(3,4-dimethoxyphenyl)hepta-1,6-diene-3,5-dione (**11**) 

To the solution of acetyl acetone (2.23 g, 22.28 mmol) in ethyl acetate, B_2_O_3_ (2.32 g, 33.43 mmol) was added and stirred at 80 °C for 30 min. The solution of dimethoxy benzaldehyde (10 g, 60.17 mmol) and tributyl borate (28.19 g, 122.58 mmol) pre-dissolved in ethyl acetate (1M) was added to the reaction mixture. After 30 min, n-butyl amine (2.44 g, 33.43 mmol) was added dropwise. After 1 h, the mixture was cooled to 50 °C and treated with the 0.5M HCl solution (100 mL) for 30 min. The reaction mixture was extracted with ethyl acetate and washed with brine and water. The organic layer was dried over anhydrous Na_2_SO_4_ and then evaporated after filtration. Recrystallization with ethanol was used to purify compound **11** (4.6 g, 52%). ^1^H NMR (CDCl_3_, 400 MHz): *δ* 7.63 (d, *J* = 15.8 Hz, 2H), 7.18–7.16 (m, 2H), 7.10 (s, 2H), 6.52 (d, *J* = 15.8 Hz, 2H), 5.84 (s, 1H), 3.98–3.90 (m, 12H); ESI-MS, *m/z*: 398.00 [M+2H]^+^.

#### 3.2.8. Synthesis of Ethyl 4-(((1E,3Z,6E)-1,7-bis(3,4-dimethoxyphenyl)-5-oxohepta-1,3,6-trien-3-yl)oxy) Butanoate (**11a**)

To the solution of compound **11** (500 mg, 1.26 mmol) in DMF (20 mL), ethyl-4-bromobutyrate (272 mg, 1.38 mmol) and K_2_CO_3_ (261 mg, 1.89 mmol) were added and stirred at room temperature. The reaction mixture was diluted with water and extracted with ethyl acetate followed by washing with brine and water. The organic layer was dried over anhydrous Na_2_SO_4_ and then evaporated after filtration. Silica gel column chromatography was used to purify compound **11a** (320 mg, 64%). ^1^H NMR (CDCl_3_, 400 MHz): *δ* 7.54 (d, *J* = 15.8 Hz, 1H), 7.10–6.96 (m, 4H), 6.82–6.75 (m, 3H), 6.43 (d, *J* = 15.7 Hz, 2H), 5.75 (s, 1H), 4.28 (t, *J* = 7.1, 14.1 Hz, 2H), 4.06–4.01 (m, 2H), 3.99–3.86 (m, 12H), 2.42 (t, *J* = 8.1, 16.3 Hz, 2H), 2.28–2.15 (m, 2H), 1.38–1.29 (m, 3H); ESI-MS, *m/z*: 511.00 [M+H]^+^.

#### 3.2.9. Synthesis of 4-(((1E,3Z,6E)-1,7-Bis(3,4-dimethoxyphenyl)-5-oxohepta-1,3,6-trien-3-yl)oxy)butanoic Acid (**11b**)

To the solution of compound **11a** (200 mg, 0.39 mmol) in THF (20 mL), LiOH (33 mg, 1.37 mmol) was added, pre-dissolved in water (2 mL), and stirred at room temperature for 12 h. The reaction mixture was concentrated in vacuo. The residue was diluted with ethyl acetate and washed with 1M HCl (50 mL). The organic layer was dried over anhydrous Na_2_SO_4_ and then evaporated after filtration. Silica gel column chromatography was used to purify compound **11b** (60 mg, 30%). ^1^H NMR (CDCl_3_, 400 MHz): *δ* 7.50 (d, *J* = 15.6 Hz, 1H), 7.08–6.99 (m, 4H), 6.82–6.76 (m, 3H), 6.67 (d, *J* = 15.7 Hz, 2H), 5.66 (s, 1H), 4.00 (t, *J* = 11.8, 5.9 Hz, 2H), 3.88–3.83 (m, 12H), 2.55 (t, *J* = 13.7, 6.9 Hz, 2H), 2.17–2.10 (m, 2H); ESI-MS, *m/z*: 483.00 [M+H]^+^.

#### 3.2.10. Synthesis of (1E,4Z)-1-(3,4-Dimethoxyphenyl)-5-hydroxyhexa-1,4-dien-3-one (**12**)

A mixture of pentane-2,4-dione (10.28 mL, 100 mmol) and boric anhydride (6.32 g, 91 mmol) in ethyl acetate (100 mL) was stirred for 30 min at 85 °C. To the mixture, the ethyl acetate solution (150 mL) of 3,4-dimethoxybenzaldehyde (6.77 mL, 45.4 mmol) and tributyl borate (5.02 mL, 18.61 mmol) were added, and then the mixture was stirred for 30 min at 85 °C. Butan-1-amine (2.92 mL, 29.5 mmol) was added dropwise to the mixture, and then the mixture was stirred for 1.5 h at 105 °C. The mixture was cooled to 50 °C and then hydrogen chloride (30.4 mL, 30.4 mmol) was added; the mixture was then stirred at the same temperature for 1 h. The resulting solution was extracted with ethyl acetate and water. The combined organic phase was dried with magnesium sulfate and filtered. After that, the solvent was concentrated in vacuo. The crude mixture was purified by MPLC (5~60% EA: 95~40% Hex) to afford the desired product (4.05 g, 36%). ^1^H NMR (400 MHz, CDCl_3_) *δ* 15.47 (s, 1H), 7.55 (d, *J* = 15.8 Hz, 1H), 7.11 (dd, *J* = 8.3, 2.0 Hz, 1H), 7.05 (d, *J* = 2.0 Hz, 1H), 6.87 (d, *J* = 8.3 Hz, 1H), 6.35 (d, *J* = 15.8 Hz, 1H), 5.65 (s, 1H), 3.93 (s, 3H), 3.92 (s, 3H), 2.16 (s, 3H); ESI-MS, *m/z*: 249.40 [M+H]^+^.

#### 3.2.11. Synthesis of *Tert*-butyl (3-(4-formyl-2-methoxyphenoxy)propyl)carbamate (**14**)

4-Hydroxy-3-methoxybenzaldehyde (2 g, 13.15 mmol), *tert*-butyl (3-bromopropyl)carbamate (4.7 g, 19.72 mmol), and potassium carbonate (2.73 g, 19.72 mmol) were dissolved in DMF (20 mL) and stirred at 80 °C for 1 h. The resulting solution was extracted with ethyl acetate and water. The combined organic phase was dried with magnesium sulfate and filtered. After that, the solvent was concentrated in vacuo. The crude mixture was purified by MPLC (10~40% EA: 90~60% Hex) to afford the desired product (3.15 g, 77%). ^1^H NMR (400 MHz, CDCl_3_) *δ* 9.86 (s, 1H), 7.48–7.38 (m, 2H), 6.96 (d, *J* = 8.1 Hz, 1H), 5.50 (br s, 1H), 4.19 (t, *J* = 5.9 Hz, 2H), 3.39 (q, *J* = 5.9 Hz, 2H), 2.07 (p, *J* = 5.9 Hz, 2H), 1.46 (s, 9H); ESI-MS, *m/z*: 309.85 [M]^+^.

#### 3.2.12. Synthesis of *Tert*-butyl (3-(4-((1E,3Z,6E)-7-(3,4-dimethoxyphenyl)-3-hydroxy-5-oxohepta-1,3,6-trien-1-yl)-2-methoxyphenoxy)propyl)carbamate (**15**)

The mixture of compound **12** (670 mg, 2.70 mmol) and boric anhydride (281 mg, 4.04 mmol) in ethyl acetate (10 mL) was stirred for 30 min at 85 °C. An ethyl acetate solution (15 mL) of compound **14** (500 mg, 1.62 mmol) and tributyl borate (1.09 mL, 4.04 mmol) was added to the mixture, and then the mixture was stirred for 30 min at 85 °C. Piperidine (0.11 mL, 1.08 mmol) was added dropwise to the mixture, and then the mixture was stirred for 1.5 h at 85 °C. The mixture was cooled to 50 °C, then hydrogen chloride (9.7 mL, 4.85 mmol) was added; the mixture was then stirred for 1 h at the same temperature. The resulting solution was extracted with ethyl acetate and water. The combined organic phase was dried with magnesium sulfate and filtered. After that, solvent was concentrated in vacuo. The crude mixture was purified by MPLC (30~90% EA: 70~10% Hex) to afford the desired product (94 mg, 11%). ^1^H NMR (400 MHz, CDCl_3_) *δ* 7.66–7.55 (m, 2H), 7.12 (dt, *J* = 8.3, 2.0 Hz, 2H), 7.06 (dd, *J* = 8.7, 2.0 Hz, 2H), 6.94 (d, *J* = 8.2 Hz, 1H), 6.89 (d, *J* = 8.3 Hz, 1H), 6.51 (d, *J* = 3.4 Hz, 1H), 6.47 (d, *J* = 3.5 Hz, 1H), 5.82 (s, 1H), 4.19–4.08 (m, 4H), 3.95 (s, 3H), 3.91 (s, 3H), 2.58–2.51 (m, 2H), 2.18 (p, *J* = 6.8 Hz, 2H), 1.29–1.23 (m, 5H); ESI-MS, *m/z*: 540.30 [M+H]^+^. 

#### 3.2.13. Synthesis of Synthesis of (1E,4Z,6E)-7-(4-(3-Aminopropoxy)-3-methoxyphenyl)-1-(3,4-dimethoxyphenyl)-5-hydroxyhepta-1,4,6-trien-3-one (**15a**)

To the solution of compound **15** (94 mg, 0.175 mmol) dissolved in MC (3 mL) was added 15% TFA in MC (2 mL) and stirred at room temperature for 30 min. The resulting solution was extracted with H_2_O. After that, solvent was concentrated in vacuo. The resulting residue was used in the next step without further purification. ESI-MS, *m/z*: 440.10 [M+H]^+^.

#### 3.2.14. Synthesis of Methyl 3-((7S,8S)-18-ethyl-3-((2-(2-(2-(4-(4-((1E,4Z,6E)-5-hydroxy-7-(4-hydroxy-3-methoxyphenyl)-3-oxohepta-1,4,6-trien-1-yl)-2-methoxyphenoxy)butanamido)et-hoxy)ethoxy)ethyl)carbamoyl)-5-(2-methoxy-2-oxoethyl)-2,8,12,17-tetramethyl-13-vinyl-7H,8H-porphyrin-7-yl)propanoate (**16**)

To the solution of compound **4** (166 mg, 0.22 mmol) dissolved in MC (3 mL) was added HBTU, O-benzotriazol-1-yl-tetramethyluronium hexafluorophosphate (125 mg, 0.33 mmol) N,N-Diisopropylethylamine (0.077 mL, 0.44 mmol) and stirred at room temperature for 20 min. Compound **8b** (100 mg, 0.22 mmol) was added to the reaction mixture and stirred at r.t for 12 h. The resulting solution was extracted with ethyl acetate and water. The combined organic phase was dried with magnesium sulfate and filtered. After that, the solvent was concentrated in vacuo. The resulting solution was purified by preparative HPLC using eluent A (0.1% TFA H_2_O) and eluent B (0.1% TFA ACN) (A/B = 70/30) (58 mg, 37%). ^1^H NMR (400 MHz, CDCl_3_) *δ* 10.06 (s, 1H), 9.99 (s, 1H), 9.20 (s, 1H), 8.07 (dd, *J* = 17.7, 11.4 Hz, 1H), 7.35 (d, *J* = 15.6 Hz, 1H), 7.25 (s, 1H), 6.99–6.81 (m, 3H), 6.78 (s, 2H), 6.50–6.15 (m, 5H), 5.72 (d, *J* = 19.2 Hz, 1H), 5.41 (d, *J* = 19.2 Hz, 1H), 4.68–4.48 (m, 3H), 4.15–4.01 (m, 1H), 3.98–3.89 (m, 4H), 3.86 (d, *J* = 11.5 Hz, 6H), 3.78–3.73 (m, 2H), 3.73–3.67 (m, 5H), 3.64–3.58 (m, 4H), 3.58–3.51 (m, 5H), 3.45–3.36 (m, 4H), 3.31–3.21 (m, 2H), 2.81–2.62 (m, 3H), 1.83–1.55 (m, 9H), 1.55–1.43 (m, 2H), 1.33–1.19 (m, 4H), 0.94–0.79 (m, 1H), −3.02 (br s, 1H); ^13^C NMR (101 MHz, CDCl_3_) *δ* 183.3, 182.8, 173.2, 172.7, 167.8, 150.0, 149.1, 147.8, 146.7, 142.4, 140.4, 139.9, 137.8, 132.4, 132.1, 128.3, 127.9, 127.6, 124.7, 122.9, 122.5, 121.9, 121.8, 114.7, 112.5, 110.2, 109.5, 101.0, 98.5, 96.8, 70.1, 69.3, 69.0, 67.8, 55.9, 55.8, 53.4, 52.4, 51.8, 49.3, 40.3, 38.3, 38.0, 31.5, 31.0, 29.5, 24.4, 23.2, 19.7, 16.5, 12.1, 12.0, 11.3; HPLC purity: 99%, retention time = 6.67 min; ESI-MS, *m/z*: 1191.35 [M]^+^. 

#### 3.2.15. Synthesis of Methyl 3-(3-(((15Z,18E)-19-(3,4-dimethoxyphenyl)-15-((E)-3,4-dimethoxystyryl)-10,17-dioxo-3,6,14-trioxa-9-azanonadeca-15,18-dien-1-yl)carbamoyl)-18-ethyl-5-(2-methoxy-2-oxoethyl)-2,8,12,17-tetramethyl-13-vinyl-7H,8H-porphyrin-7-yl)propanoate (**17**)

To the solution of compound **11b** (300 mg, 0.622 mmol) and compound **4** (516 mg, 0.684 mmol) in CH_2_Cl_2_ (60 mL), EDCI (239 mg, 1.25 mmol), HOBt (168 mg, 1.25 mmol), and DIPEA (241 mg, 1.86 mmol) were added and stirred at room temperature overnight. The reaction was quenched with water and extracted with CH_2_Cl_2_. The mixture was washed with brine and water. The organic layer was dried over anhydrous Na_2_SO_4_ and then evaporated after filtration. Silica gel column chromatography was used to purify compound **17** (280 mg, 38%). ^1^H NMR (400 MHz, CDCl_3_): *δ* 9.71 (s, 1H), 9.60 (s, 1H), 8.79 (s, 1H), 8.06–7.98 (m, 2H), 7.46 (d, *J* = 15.7 Hz, 1H), 7.17–7.02 (m, 5H), 6.98–6.86 (m, 3H), 6.53 (d, *J* = 15.7 Hz, 1H), 6.33 (d, *J* = 17.9 Hz, 1H), 6.10 (d, *J* = 11.2 Hz, 1H), 5.65 (d, *J* = 17.7 Hz, 1H), 4.41 (dd, *J* = 37.2 Hz, 8.3 Hz, 2H), 4.09–4.08 (m, 1H), 3.97–3.91 (m, 13H), 3.84–3.76 (m, 6H), 3.72–3.60 (m, 8H), 3.55–3.50 (m, 2H), 3.42 (s, 3H), 3.32 (s, 3H), 3.13–3.08 (m, 2H), 2.75–2.55 (m, 4H), 2.53–2.16 (m, 2H), 1.81–1.72 (m, 6H), 1.27 (s, 1H), 0.90–0.71 (m, 4H), −1.61 (s, 1H), −1.77 (s, 1H); ^13^C NMR (101 MHz, CDCl_3_) *δ* 187.5, 174.2, 173.4, 171.6, 169.5, 168.9, 166.6, 166.0, 154.4, 150.8, 150.1, 149.1, 144.8, 140.3, 139.0, 136.3, 136.14, 135.0, 134.7, 134.6, 130.4, 129.6, 129.3, 129.3, 128.2, 128.1, 127.2, 122.7, 122.0, 121.7, 119.36, 110.9, 110.9, 109.5, 102.2, 101.3, 100.5, 98.9, 93.6, 70.2, 69.5, 69.4, 66.4, 55.9, 55.9, 55.8, 53.0, 52.2, 51.6, 49.1, 40.3, 38.6, 38.5, 37.7, 31.0, 30.7, 29.7, 23.2, 23.1, 19.7, 17.7, 12.1, 11.9, 11.3; HPLC purity: 99%, retention time = 6.81 min; ESI-MS, *m/z*: 1220.00 [M+H]^+^.

#### 3.2.16. Synthesis of Methyl 3-((7S,8S)-18-ethyl-3-((16-(4-((1E,4Z,6E)-5-hydroxy-7-(4-hydroxy-3-methoxy-phenyl)-3-oxohepta-1,4,6-trien-1-yl)-2-methoxyphenoxy)-12-oxo-3,6,9-trioxa-13-azahexadecyl)carbamoyl)-5-(2-methoxy-2-oxoethyl)-2,8,12,17-tetramethyl-13-vinyl-7H,8H-porphyrin-7-yl)propanoate (**18**)

To the solution of compound **6** (118 mg, 0.14 mmol) dissolved in MC (5 mL) was added HBTU, O-benzotriazol-1-yl-tetramethyl-uronium hexafluorophosphate (91 mg, 0.21 mmol), and N,N-Diisopropylethylamine (0.050 mL, 0.28 mmol) and stirred at room temperature for 20 min. Compound **9b** (91 mg, 0.21 mmol) was added in the reaction mixture and stirred at r.t for 12 h. The resulting solution was extracted with ethyl acetate and water. The combined organic phase was dried with magnesium sulfate and filtered. After that, the solvent was concentrated in vacuo. The resulting solution was purified by preparative HPLC using eluent A (0.1% TFA H_2_O) and eluent B (0.1% TFA ACN) (A/B = 70/30) (51 mg, 29%). ^1^H NMR (400 MHz, CDCl_3_) *δ* 9.97 (s, 1H), 9.90 (s, 1H), 9.09 (s, 1H), 8.05 (br s, 1H), 7.98 (dd, *J* = 17.8, 11.5 Hz, 1H), 7.43 (d, J = 15.5 Hz, 1H), 7.35 (d, *J* = 15.2 Hz, 1H), 7.04 (d, *J* = 8.1 Hz, 1H), 6.93–6.87 (m, 3H), 6.76 (br s, 1H), 6.56 (d, *J* = 7.7 Hz, 1H), 6.36–6.21 (m, 3H), 6.16 (d, *J* = 15.5 Hz, 1H), 5.64 (d, *J* = 18.8 Hz, 1H), 5.42 (br s, 1H), 5.34 (d, *J* = 18.8 Hz, 1H), 4.58 (dd, *J* = 13.9, 6.7 Hz, 1H), 4.51 (d, *J* = 10.0 Hz, 1H), 4.16–4.02 (m, 2H), 4.00–3.93 (m, 2H), 3.93–3.86 (m, 3H), 3.85 (s, 3H), 3.78 (s, 5H), 3.72 (s, 3H), 3.71–3.66 (m, 3H), 3.64 (s, 3H), 3.51 (br s, 2H), 3.47 (s, 5H), 3.37 (s, 4H), 3.12 (br s, 2H), 2.77–2.59 (m, 3H), 2.37–2.28 (m, 1H), 2.26–2.15 (m, 1H), 2.00 (s, 1H), 1.82 (d, *J* = 7.1 Hz, 3H), 1.79–1.61 (m, 3H), 1.62–1.50 (m, 5H), 1.33–1.19 (m, 3H), 0.93–0.79 (m, 1H), −2.16 (br s, 1H), −2.78 (br s, 1H); HPLC purity: 99%, retention time = 6.72 min; ESI-MS, *m/z*: 1235.25 [M]^+^.

#### 3.2.17. Synthesis of Methyl 3-((7S,8S)-3-((16-(4-((1E,3Z,6E)-7-(3,4-dimethoxyphenyl)-3-hydroxy-5-oxohepta-1,3,6-trien-1-yl)-2-methoxyphenoxy)-12-oxo-3,6,9-trioxa-13-azahexadecyl)carbamoyl)-18-ethyl-5-(2-methoxy-2-oxoethyl)-2,8,12,17-tetramethyl-13-vinyl-7H,8H-porphyrin-7-yl)propanoate (**19**)

To the solution of compound **6** (132 mg, 0.16 mmol) dissolved in MC (5 mL) was added HBTU, O-benzotriazol-1-yl-tetramethyl-uronium hexafluorophosphate (91 mg, 0.24 mmol), and N,N-Diisopropylethylamine (0.056 mL, 0.32 mmol) and stirred at room temperature for 20 min. Compound **15a** (132, 0.16 mmol) was added to the reaction mixture and stirred at room temperature for 12 h. The resulting solution was extracted with ethyl acetate and water. The combined organic phase was dried with magnesium sulfate and filtered. After that, the solvent was concentrated in vacuo. The resulting solution was purified by preparative HPLC using eluent A (0.1% TFA H_2_O) and eluent B (0.1% TFA ACN) (A/B = 70/30) (51 mg, 26%). ^1^H NMR (400 MHz, CDCl_3_) *δ* 9.95 (s,1H), 9.89 (s, 1H), 9.05 (s, 1H), 8.14–7.88 (m, 2H), 7.48 (d, *J* = 15.5 Hz, 1H), 7.37 (d, *J* = 15.5 Hz, 1H), 7.09 (d, *J* = 8.2 Hz, 1H), 7.03–6.76 (m, 5H), 6.61 (d, *J* = 7.9 Hz, 1H), 6.37–6.15 (m, 4H), 5.64–5.47 (m, 2H), 5.30 (d, *J* = 19.0 Hz, 1H), 4.54 (dt, *J* = 26.9, 8.6 Hz, 2H), 4.05 (s, 1H), 3.93 (br s, 3H), 3.88 (s, 6H), 3.81–3.66 (m, 12H), 3.62 (br s, 2H), 3.52–3.40 (m, 7H), 3.36 (s, 5H), 3.18–3.01 (m, 2H), 2.74 (br s, 2H), 2.67–2.55 (m, 1H), 2.37–2.25 (m, 1H), 2.21–1.96 (m, 2H), 1.90–1.69 (m, 5H), 1.67–1.46 (m, 6H), 1.26 (s, 6H), 0.93–0.69 (m, 2H), −1.89 (br s, 1H), −2.53 (br s, 1H); ^13^C NMR (101 MHz, CDCl_3_) *δ* 183.2, 182.9, 173.2, 172.7, 171.2, 151.0, 149.9, 149.2, 149.1, 143.3, 142.6, 140.9, 140.3, 140.0, 139.6, 138.2, 136.7, 135.4, 134.3, 132.6, 132.3, 128.2, 128.0, 125.0, 122.6, 122.5, 122.0, 121.9, 112.4, 111.1, 110.1, 109.7, 105.2, 101.0, 98.8, 96.3, 70.2, 70.0, 69.9, 69.6, 67.2, 66.5, 56.0, 55.8, 55.7, 53.0, 52.5, 51.8, 49.1, 40.2, 38.1, 36.9, 35.6, 30.8, 29.7, 29.2, 28.1, 23.1, 19.7, 16.5, 12.0, 11.3; HPLC purity: 99%, retention time = 7.37 min; ESI-MS, *m/z*: 1249.20 [M]^+^.

### 3.3. Photophysical Properties and Singlet Oxygen Photogeneration Analysis

The UV–vis spectra of Ce6 and Ce6-curcumin derivative were analyzed in DMSO (Duksan, HPLC grade) at a concentration of 10 µM (Thermo-scientific, Skanlt software 5.0, Waltham, MA, USA). The data were corrected for solvent background by the instrument’s calibration using DMSO as a blank. The absorption spectra of the samples were obtained using cuvette with a 1 cm path length in the range of 300–800 nm at a 1 nm interval. The fluorescence intensities of Ce6 and Ce6-curcumin derivative were recorded on a Spark^®^ multimode microplate reader (Tecan Trading AG, Männedorf, Switzerland) at the emission wavelength range of 500–800 nm. The samples prepared in DMSO at the concentration of 20 µM were kept in a 96-well plate and fluorescence intensity was analyzed using a excitation wavelength of 405 nm at a 1 nm interval.

1, 3-Diphenylisobenzofuran (DPBF) was used as a selective ^1^O_2_ acceptor, which was bleached upon reaction with ^1^O_2_. The sample solutions of DPBF in DMSO (50 μM) containing, respectively, DPBF only (50 μM, as a control), DPBF + curcumin (1, 5, and 10 μM), DPBF + MB (1, 5, and 10 μM), DPBF + Ce6 (1, 5, and 10 μM), and DPBF + **17** (1, 5, and 10 μM), were prepared in the dark. All the samples were placed in a 96-well plate and the container was covered with aluminum foil. The samples were irradiated (660 nm, 50 mW, 2 J/cm^2^) for 8 min and 20 s. After irradiation, the visible spectra of the sample solutions were measured spectrophotometrically. The normalized absorbance of DPBF at 418 nm in these samples was reported. The ^1^O_2_ photo-generation activities of curcumin, MB, Ce6, and **17** can be compared with the different absorbance decay of each sample relative to the DPBF control sample. 

### 3.4. Biological Analysis

#### 3.4.1. Measurement of Cellular Uptake 

The degree of the cellular absorption of Ce6 and its derivatives was assessed using flow cytometry techniques. AsPC-1, MIA-PaCa-2, and PANC-1 cells were seeded into the 6-well plates at the density of 2 × 10^5^ cells per well, respectively, and incubated overnight. The culture medium was replaced and various synthesized compounds were treated at the final concentration of 5 μΜ into respective wells for 3 h. The cells were then washed thrice with phosphate-buffered saline (PBS), centrifuged, and re-suspended in 0.5 mL of PBS. The fluorescence intensity of cells was accessed by using FACS (Cytomics™ FC 500, Beckman Coulter, Miami, FL, USA).

#### 3.4.2. Cell Viability Assay 

AsPC-1, the human pancreatic cancer cells obtained from the Korean Cell Line Bank (KCLB, Seoul, Republic of Korea), were grown in the RPMI-1640 medium containing 10% fetal bovine serum and 1% penicillin. MIA-PaCa-2 and PANC-1 cells procured from the Korean Cell Line Bank (KCLB, Seoul, Republic of Korea) were grown in DMEM supplemented with 10% fetal bovine serum, 1% penicillin, and streptomycin, which were then kept in an atmosphere of 5% CO_2_ humidified at 37 °C. The respective cell lines were seeded in 96-well plates and treated with or without different concentrations of the synthesized compounds for 3 h. The 3 h incubation time was taken based on our previous study. The cells were irradiated with a laser of 660 nm, 50 mW, 5 J/cm^2^ along with the different doses of the synthesized compounds. For the evaluation of the dark toxicity of the synthesized compounds, the cells were kept under similar conditions without laser irradiation. After 72 h of incubation, cell viability was measured using the 4,5-dimethylthiazol-2-yl)-2,5-diphenyl tetrazolium bromide (MTT) assay, and the absorbance was measured at 540 nm using a microplate reader (Multiskan GO, Thermo Scientific, Waltham, MA, USA).

#### 3.4.3. Annexin V and Propidium Iodide Staining

Apoptotic cells were measured by using the FITC Annexin V apoptosis detection kit (BD Biosciences, San Jose, CA, USA). Briefly, AsPC-1, MIA-PaCa-2, and PANC-1 cells were seeded at a density of 2 × 10^5^ cells/mL in 6-well plates. Cells were pre-treated with **17** for 3 h prior to irradiation with the light (660 nm, 50 mW, 5 J/cm^2^), and then were further incubated for 72 h. The cells were then collected, washed three times with cold 1X PBS, and further suspended in the FACS buffer. Annexin V and PI staining were then carried out as per the manufacturer’s protocol.

#### 3.4.4. Western Blot 

Briefly, cells were seeded in 6-well plates and treated with **17**. After 3 h, cells were irradiated with the laser light (660 nm, 50 mW, 5 J/cm^2^) and incubated for an additional 24 h. Total protein was isolated from the AsPC-1, MIA-PaCa-2, and PANC-1 cells by using the RIPA lysis buffer (150 mM sodium chloride, 1% Triton X-100, 0.5% sodium deoxycholate, 0.1% SDS, and 50 mM Tris adjusted to pH 8.0) containing 1X proteases and phosphatases inhibitors for 1 h. The lysates were centrifuged at 10,000× *g* for 10 min at 4 °C, and the supernatant fractions were collected. Equal amounts of proteins from the samples were separated using 10% SDS–polyacrylamide gel electrophoresis (SDS-PAGE) and electro-transferred in Immobilon P membranes (Millipore Corp., Bedford, MA, USA). The membranes were incubated at 4 °C overnight with different primary antibodies: anti-mouse anti-Bcl-2 (Bioss, bs-52022M), anti-rabbit β-Actin (abcam, 8226) antibodies, and cytochrome c (Bioss, bs-0013R). The results were visualized using an enhanced chemiluminescence (ECL) reagent and the images were taken using a luminescent image analyzer (Amersham, GE Healthcare, Piscataway, NJ, USA). 

#### 3.4.5. Animal Model

All the mouse experiments were reviewed and carried out in approval of an Institutional Animal Care and Use Committee of the Dongsung Cancer Center under protocol IACUC #ds002106117-2. C57BL/6 mice (*n* = 16) and ICR mice (*n*= 36) aged six weeks were obtained from Orient Bio (Sungnam, South Korea) and maintained at 28 °C under pathogen-free conditions in a controlled animal house facility of Dongsung Cancer Center, Daegu, for seven days with an alternate light and dark cycle of 12 h. Each experimental group consisted of randomly grouped mice of the same weight. 

#### 3.4.6. Xenograft Mouse Model Using B16F10 Melanoma Cell

For tumor inoculation, 1 × 10^4^ cells in 0.1 mL media/matrigel (1:1, *v/v*) were injected subcutaneously in the right flank of the mice and monitored daily. Subcutaneous tumors induced by B16F10 cells in C57BL/6 mice were randomly divided into control, laser only, **17** only, and **17**-PDT-treated groups (4 in each group).

#### 3.4.7. PDT in Animal Model

After the tumor volume had reached the size of 100 mm^3^, the mice were injected intravenously with normal saline and 2.5 mg/kg of the **17** solution in the control and treatment groups, respectively. The 2.5 mg/kg dose of **17** for the in vivo analysis was chosen according to our previously published study [50]. After this, the tumors on the right side were exposed to a 660 nm laser diode, (LD, LEMT, Belarus) at a dose of 100 J/cm^2^ (irradiation fluence rate 200 mW/cm^2^, irradiation time 500 s) under anesthesia, 3 h after drug administration. The tumor volume was recorded every 2 days up to 14 days after tumor inoculation. The tumor volume (v) was measured with Vernier caliper and calculated as v = l × w^2^/2, where “l” and “w” were the length and width of the tumor, respectively. Experimental animals were euthanized on the 14th day post-inoculation when the tumor volume of the untreated group exceeded 1500 mm^3^.

#### 3.4.8. Pharmacokinetics

ICR mice (6 weeks of age) were intravenously administered with either **17** (2.5 mg/kg) or the vehicle (10% DMSO, 10% Tween 80, and 80% water). The mice were anaesthetized 5–10 min before the collection of blood. Blood samples were drawn from the lateral tail vein at 1, 2, 3, 4, 5, and 6 h after dosing and were then centrifuged at 10,000× *g* for 5 min. The fluorescence intensity was measured spectrophotometrically at 660 nm using a Spark Multimode Microplate Reader (Tecan Trading AG, Männedorf, Switzerland) based on the concentration of **17** in blood plasma.

#### 3.4.9. Animals and Treatment

Four groups of female ICR mice (*n* = 3) received intravenous injections of low and high doses of Ce6 (2.5 and 5 mg/kg), or none. The survival and behavior of the animals were then assessed 1 to 10 days after the injection. At the same time, three control groups of female mice (*n* = 3) were injected with the vehicle (10% DMSO, 10% Tween 80, and 80% water). Animal weights were assessed to determine the body weight loss due to **17**. Furthermore, the mice were also monitored for their daily food and water intake. We also identified the various signs and symptoms, such as mortality, convulsions, aggressiveness, respiration, strange behaviors, skin scarring, etc.

### 3.5. Statistical Analysis

The data are presented as the mean  ±  standard error of the mean. All statistical analyses were performed using GraphPad Prism software (v5.02; La Jolla, CA, USA) by using one-way ANOVA with Tukey’s post hoc test for multiple comparisons. A *p*-value  <  0.05 was considered statistically significant. 

## 4. Conclusions

A total of four novel Ce6-curcumin derivatives were synthesized and characterized by NMR and ESI-MS. Among them, **17** showed excellent cellular uptake and singlet oxygen generation capability as compared to the control (Ce6). All the synthesized compounds were evaluated for their PDT efficacy in vitro and in vivo. **17**, with remarkable cellular internalization capability, showed significant cytotoxicity against AsPC-1 (0.27 µM), MIA PaCa-2 (0.42 µM), and PANC-1 (0.21 µM) cell lines, which is 101.18, 26.26, and 89.19 times more photo-toxic, respectively, as compared to dark toxicity. Similarly, **17** caused cancer cell death by intrinsic apoptosis in all tested cell lines through the downregulation of Bcl-2 and upregulation of the cytochrome C protein expression. Structure–activity relationship studies have revealed that the incorporation of additional methyl ester moieties and the conjugation to the enone moiety of curcumin is crucial for displaying better cellular uptake and PDT efficacy. Moreover, the in vivo PDT efficacy study of **17** suggested its potential to significantly lower the tumor growth in the B16F10 melanoma mouse model. These positive results in vivo must be due to the maximal blood concentration of **17** post 4 h of injection and the absence of toxicity. Thus, this study might provide valuable information for the researchers working on Ce6 derivatives as a potential PS having enhanced PDT efficacy in vitro and in vivo.

## Figures and Tables

**Table 1:** The dark and photo cytotoxicity of synthesized compounds.

Compounds	Dark Toxicity (IC_50_, µM)			Phototoxicity (IC_50_, µM)			Ratio * (AsPC-1, MIA-PaCa-2, PANC-1)
	AsPC-1	MIA-PaCa-2	PANC-1	AsPC-1	MIA-PaCa-2	PANC-1	
**I**	≥50	≥50	≥50	0.04	0.035	0.04	ND, ND, ND
**II**	≥50	≥50	≥50	≥3.1	≥3.1	≥3.1	ND, ND, ND
**16**	≥50	≥50	≥50	≥3.1	2.05	2.15	ND, ND, ND
**17**	27.32	11.03	18.73	0.27	0.42	0.21	101.18, 26.26, 89.19
**18**	≥50	≥50	≥50	≥3.1	2.33	2.41	ND, ND, ND
**19**	≥50	≥50	24.4	≥3.1	2.19	2.03	ND, ND, 12.01
**Ce6**	≥50	≥50	≥50	≥3.1	≥3.1	≥3.1	ND, ND, ND

* Dark to phototoxicity ratio against tested cell lines; ND: not determined.

**Table 2:** The apoptotic percentage induced by **17**-PDT in tested cell lines.

Cell Line	Concentration (µM)	Q1 (NE)	Q2 (LA)	Q3 (Live)	Q4 (EA)	Q2 + Q4
**AsPC-1**	Control	3.51%	2.83%	91.51%	2.15%	4.98%
	0.2	2.67%	50.65%	41.34%	5.34%	55.99%
	0.4	4.39%	58.59%	27.30%	9.72%	68.31%
	0.8	0.10%	84.35%	7.92%	7.63%	91.98%
**MIA-PaCa-2**	Control	0.15%	0.12%	99.11%	0.62%	0.74%
	0.1	7.94%	52.17%	29.91%	9.98%	62.15%
	0.2	7.70%	46.77%	34.70%	10.83%	57.60%
	0.4	0.24%	57.85%	7.81%	34.10%	91.95%
**PANC-1**	Control	26.09%	3.00%	69.94%	0.97%	3.97%
	0.1	10.09%	21.21%	42.76%	25.94%	47.15%
	0.2	5.37%	26.83%	38.49%	29.31%	56.14%
	0.4	1.25%	31.96%	13.41%	53.38%	85.34%

Note: Q1 = necrosis (NE); Q2 = late apoptosis (LA); Q3 = live; and Q4 = early apoptosis (EA).

## Data Availability

All the data are contained within the manuscript and supplementary materials.

## Supplementary Materials

The following supporting information can be downloaded at: https://www.mdpi.com/article/10.3390/pharmaceutics15061577/s1, Section S1: ^1^H & ^13^C NMR spectra of selected compounds; Section S2: ESI-MS spectra of selected compounds; Section S3: Binding affinity of synthesized compounds **16**, **18**, and **19** to LDL; and Section S4: Signs and symptoms recorded from day 1–10 after 17 was injected into the ICR mice.
