# Supplementary material for: Novel Chlorin e6-Curcumin Derivatives as a Potential Photosensitizer: Synthesis, Characterization, and Anticancer Activity
Source: Pharmaceutics. 2023 May 23;15(6):1577. doi: 10.3390/pharmaceutics15061577 (PMC10300926; doi:10.3390/pharmaceutics15061577)
Supplement: Supplementary file 1 [file pharmaceutics-15-01577-s001.zip › pharmaceutics-2374901-supplementary.pdf]

Novel chlorin e6-curcumin derivatives as a potential photosensitizer: Synthesis, characterization, and anticancer activity

Table of contents:

S1. ^{1}H and ^{13}C NMR spectra of selected compounds

S2. Mass spectra of selected compounds

S3. Binding affinity of synthesized compounds **16**, **18**, and **19** to LDL

S4. Signs and symptoms recorded from day 1-10 after **17** was injected into the ICR mice

S1. ^{1}H NMR spectra of selected compounds

*1.1. ^{1}H NMR spectrum of compound **11***

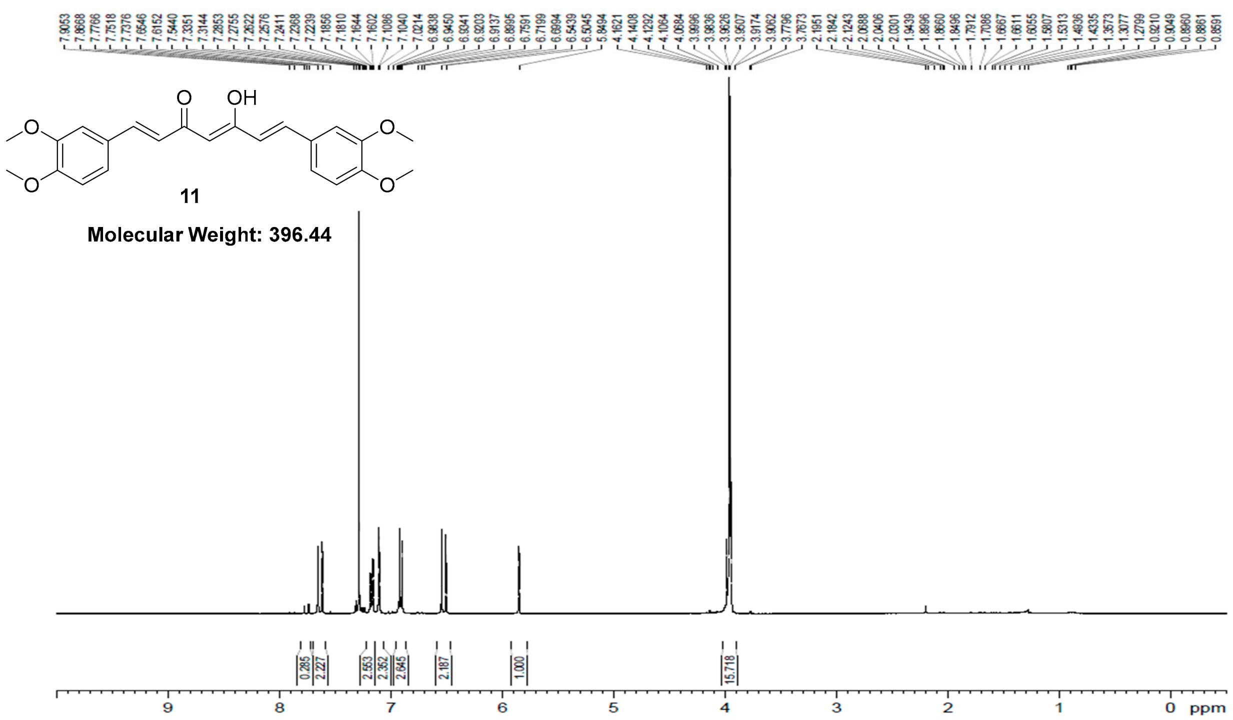

*1.2. ^{1}H NMR spectrum of compound **11a***

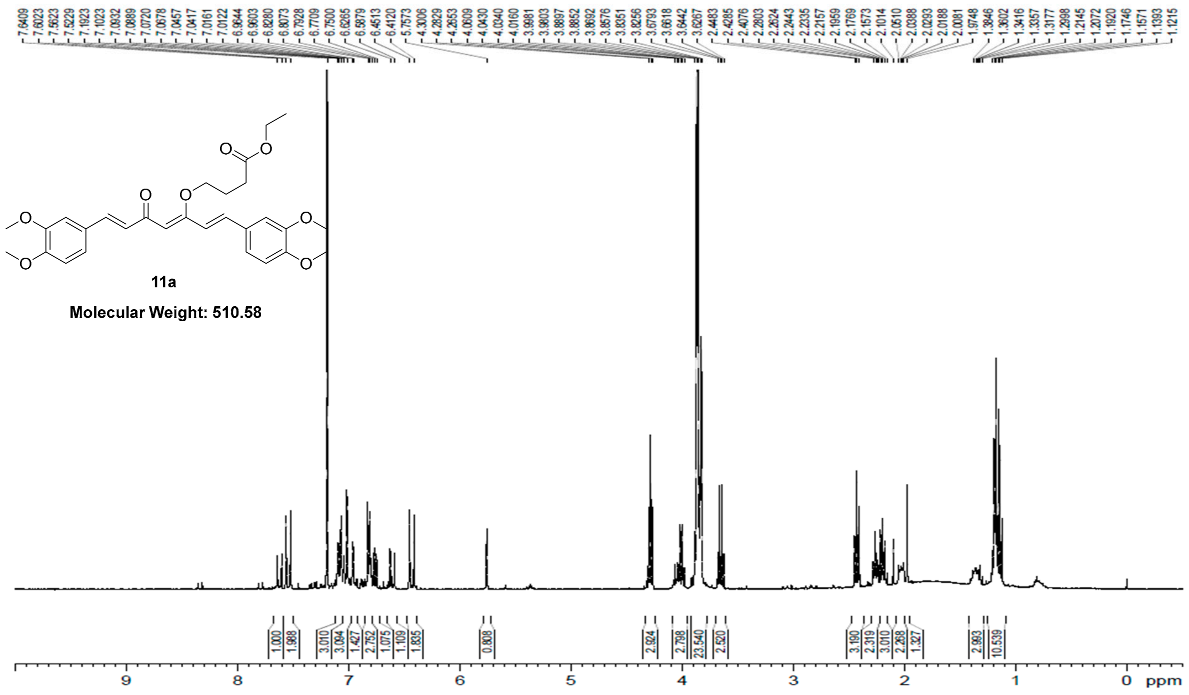

1.3. ^{1}H NMR spectrum of compound ***11b***

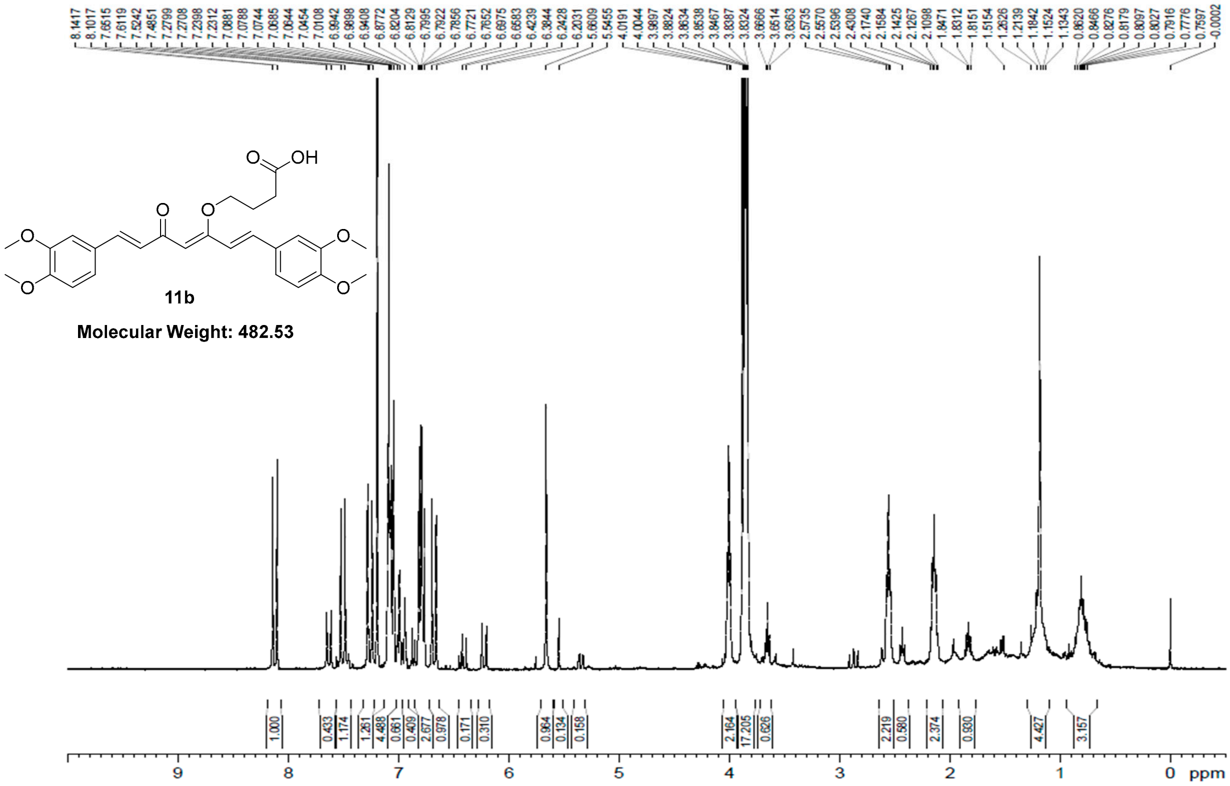

*1.4. ^{1}H NMR spectrum of compound **16***

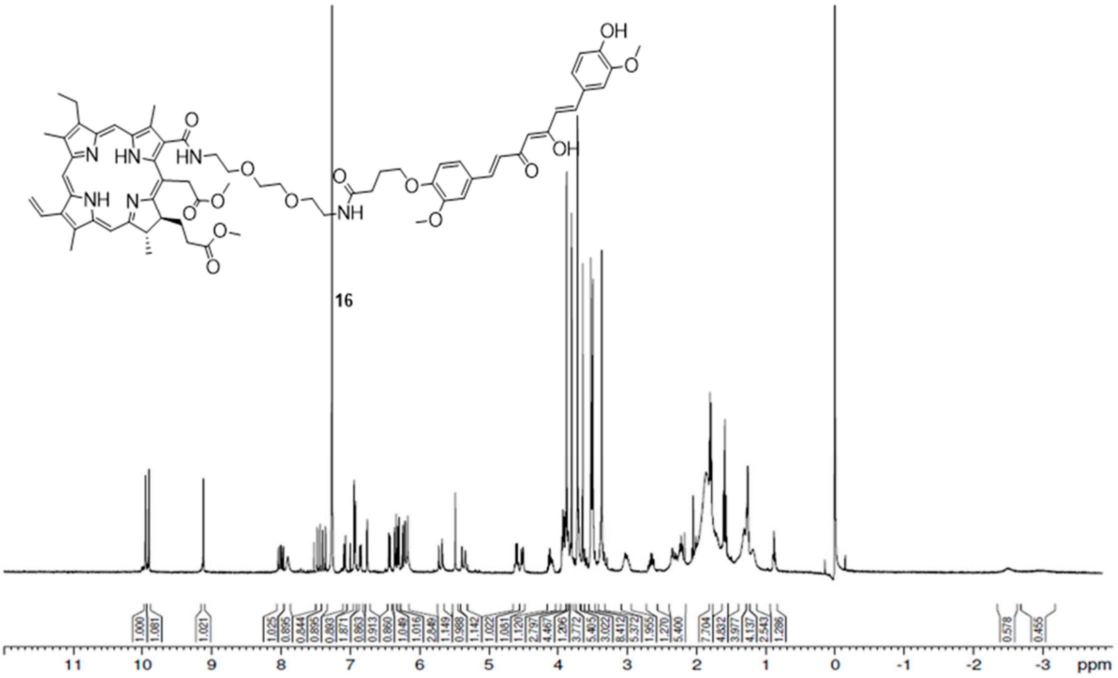

*1.5. ^{13}C NMR spectrum of compound **16***

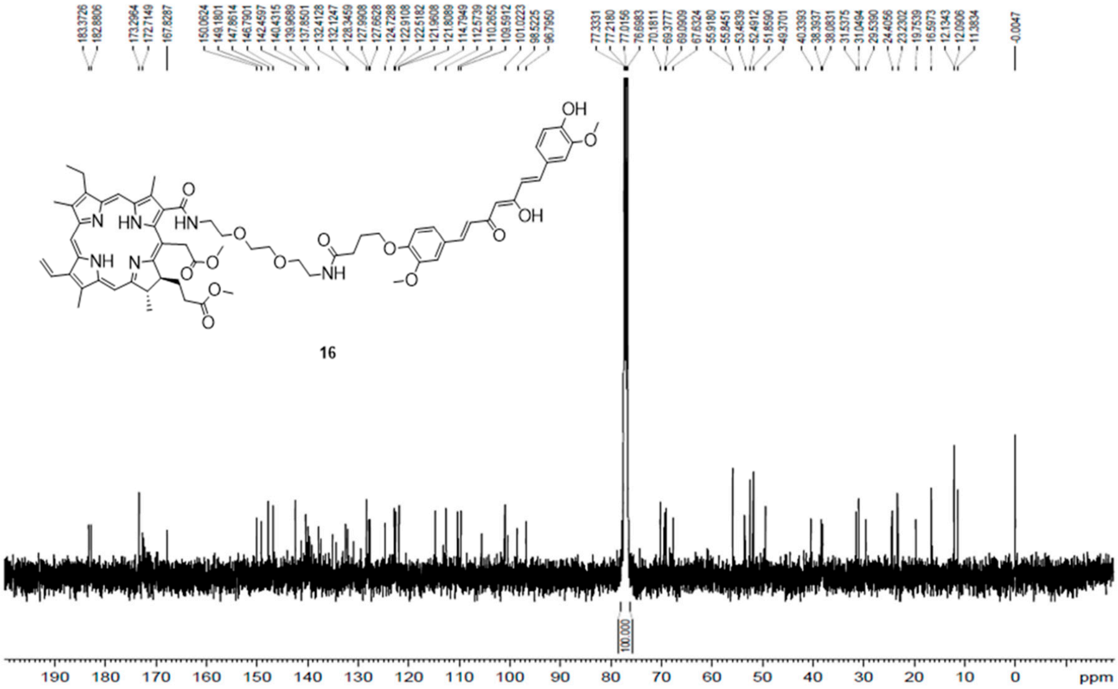

1.6. ^{1}H NMR spectrum of compound **17**

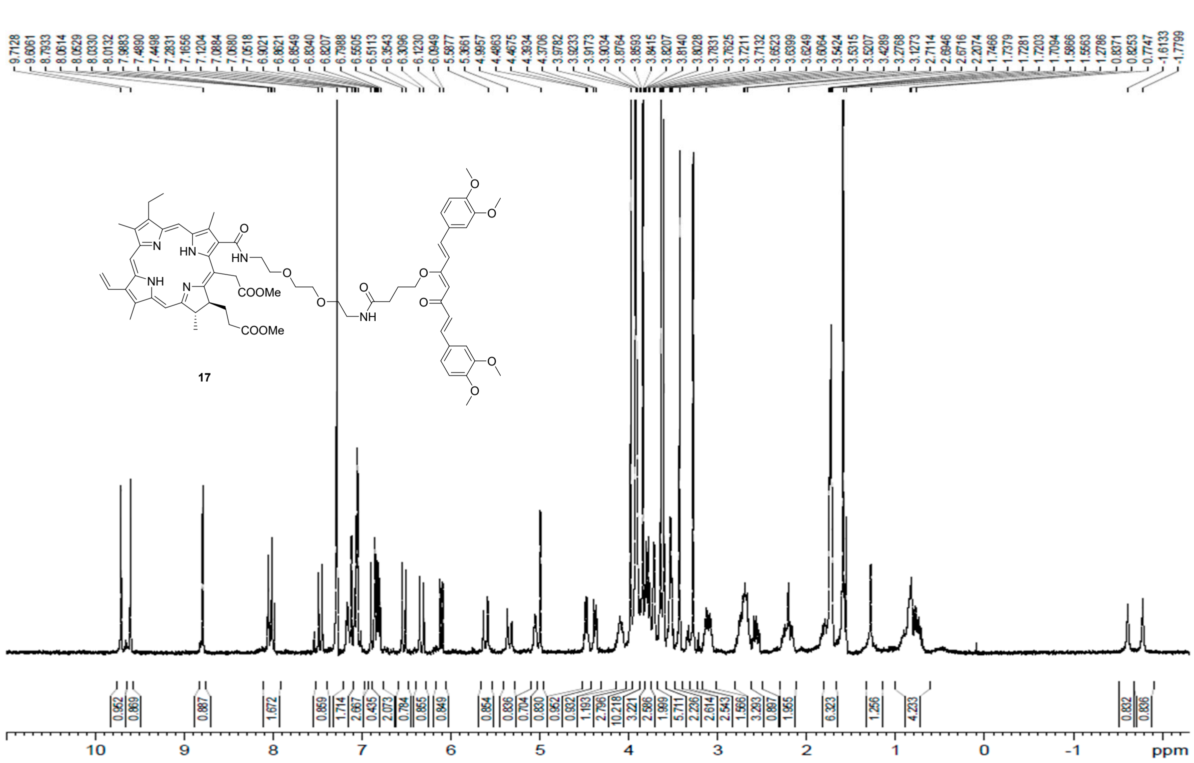

1.7. ^{13}C NMR spectrum of compound **17**

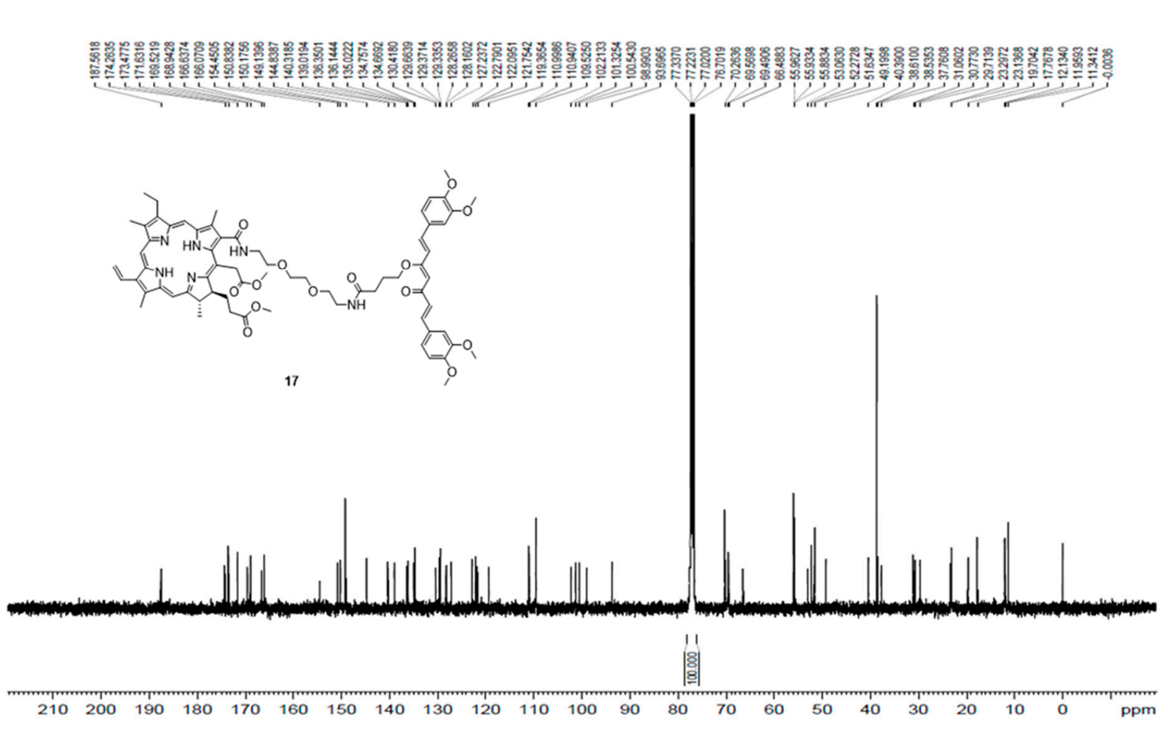

*1.8. ^{1}H NMR spectrum of compound **18***

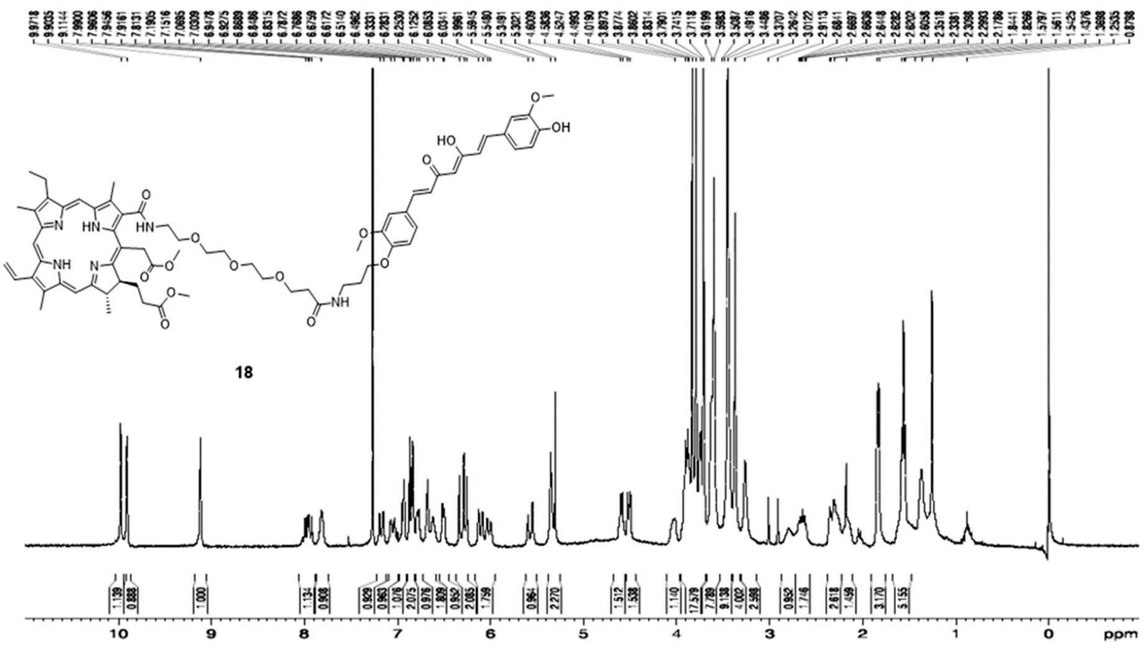

*1.9. ^{1}H NMR spectrum of compound **19***

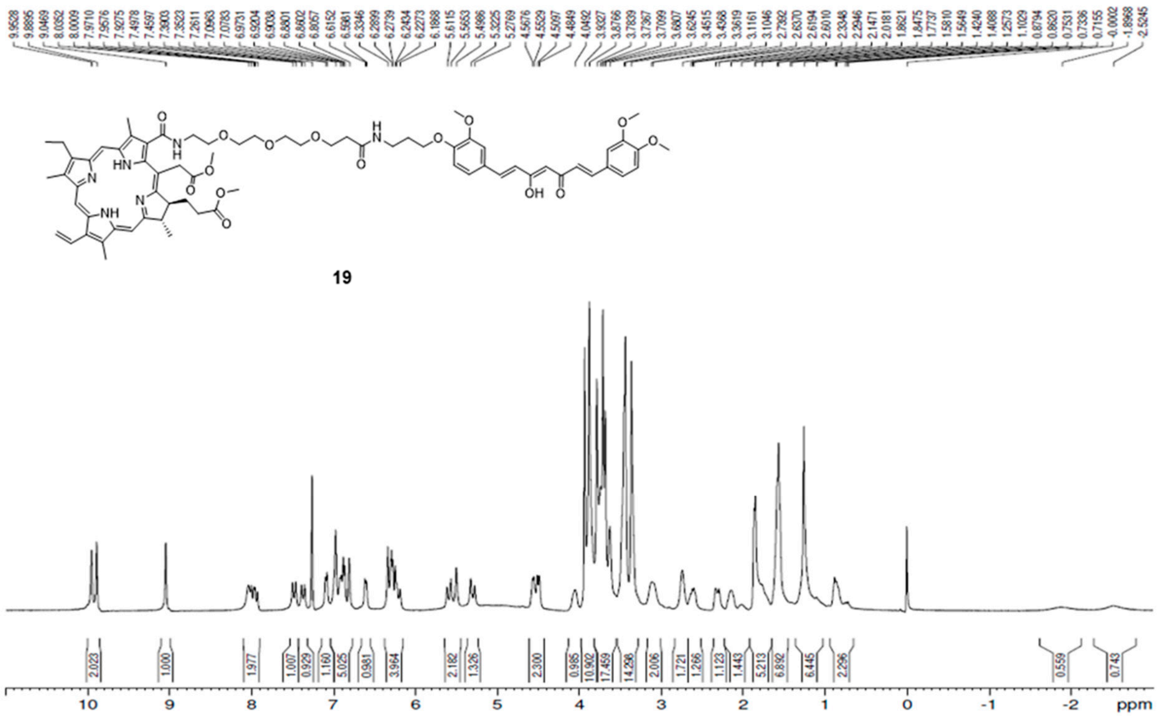

*1.10. ^{13}C NMR spectrum of compound **19***

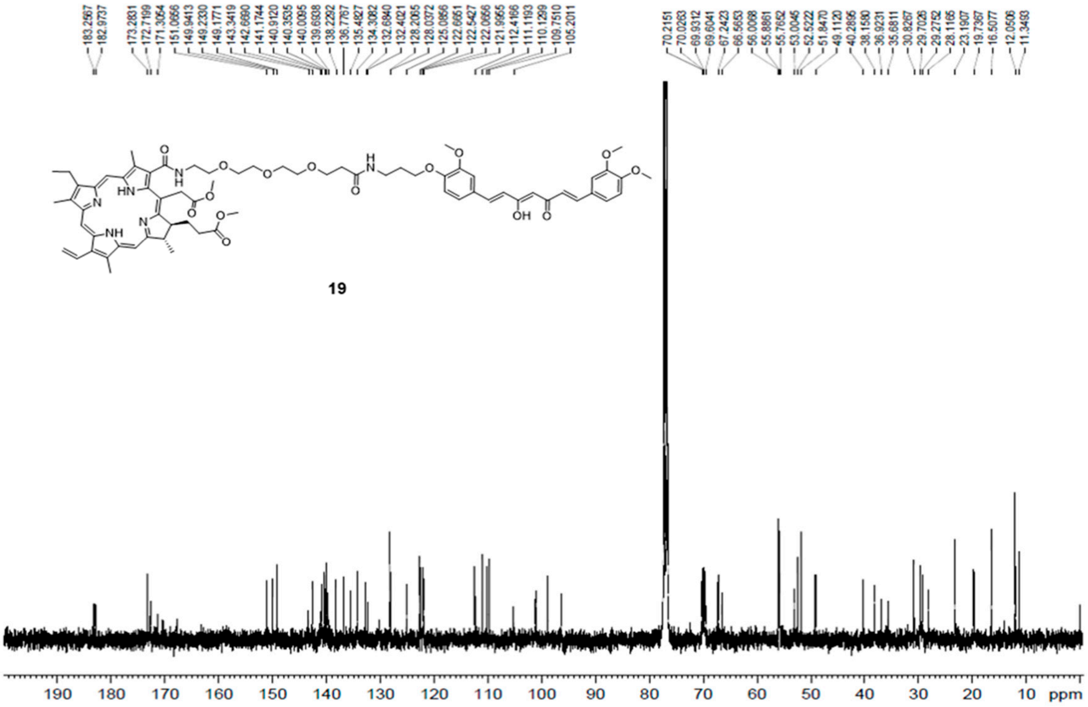

S2. Mass spectra of selected compounds

*2.1. Mass spectrum of compound **11***

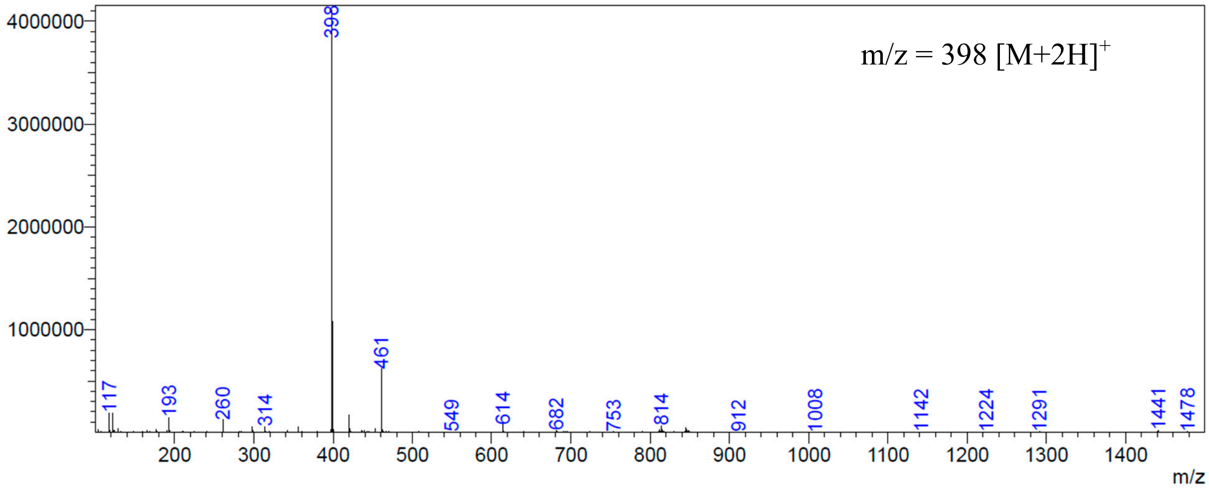

2.2. Mass spectrum of compound ***11a***

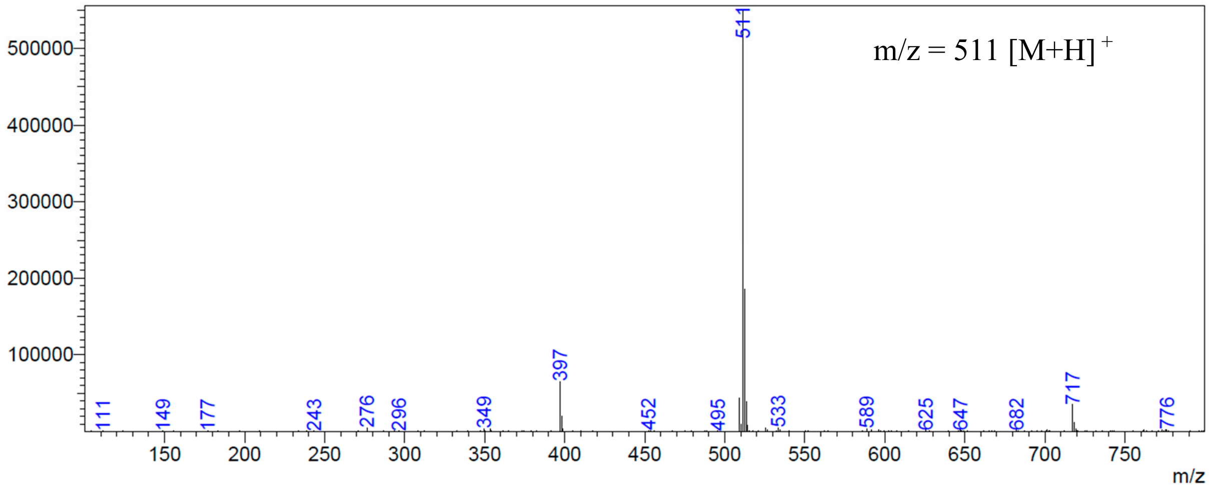

2.3. Mass spectrum of compound ***11b***

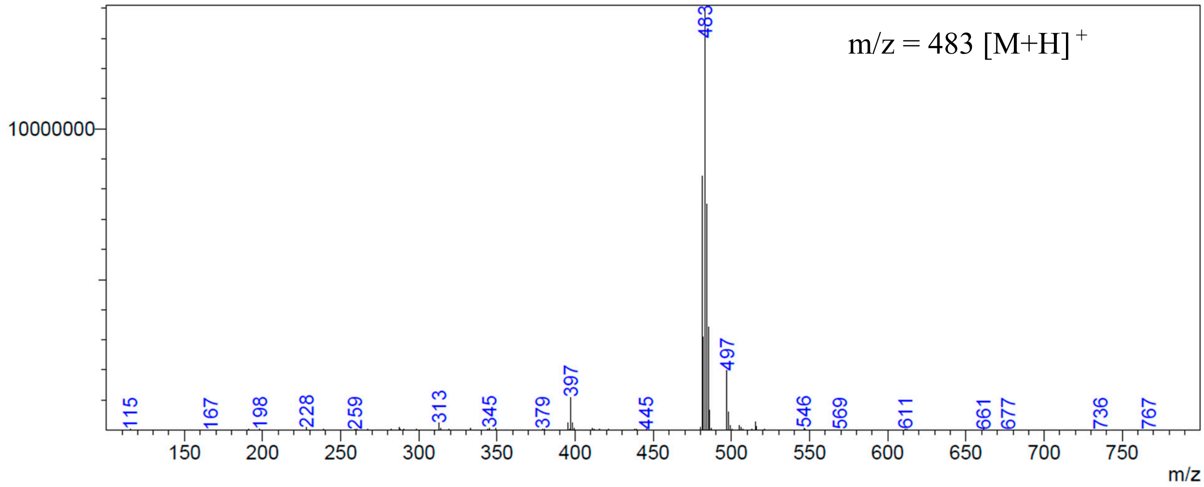

*2.4. Mass spectrum of compound **17***

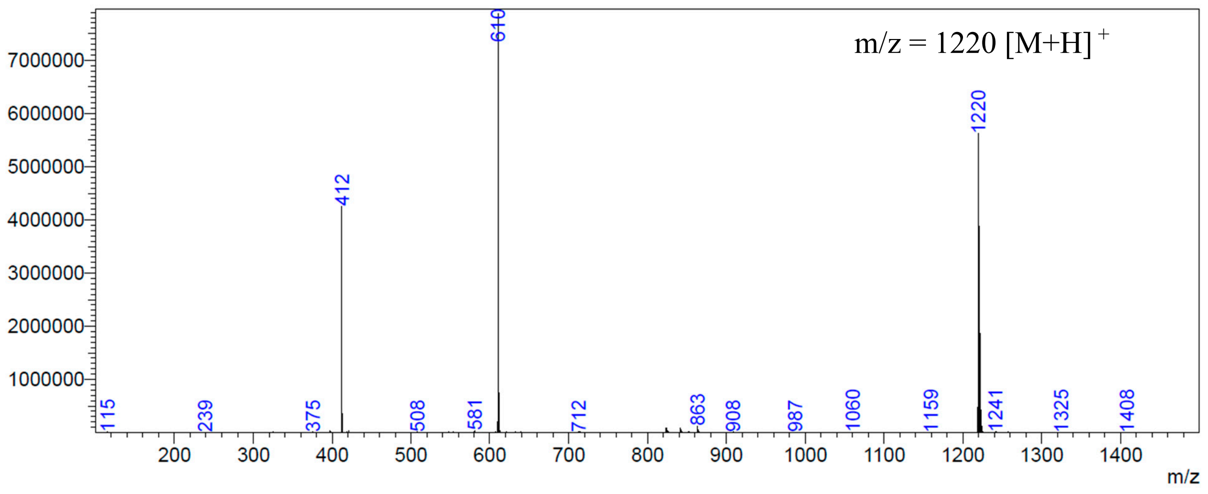

S3. Binding affinity of synthesized compounds 16, 18, and 19 to LDL

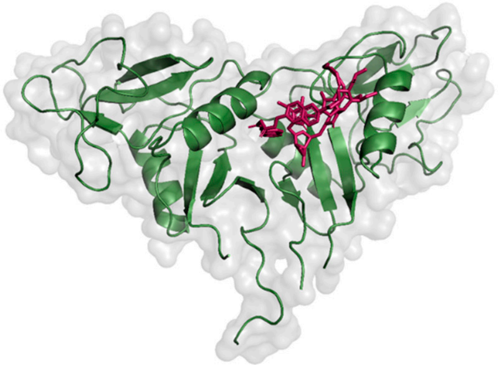

LBE: -7.3 kcal/mol
Pocket volume: 1042 Å^3
Compound **16** (——)

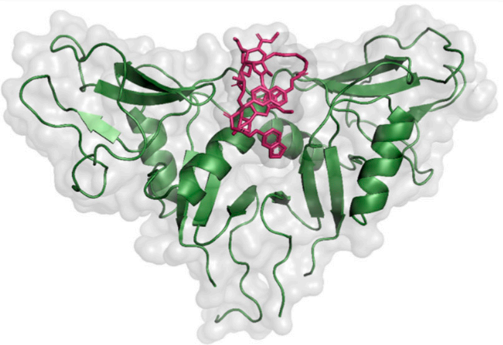

LBE: -6.8 kcal/mol
Pocket volume: 1042 Å^3
Compound **18** (——)

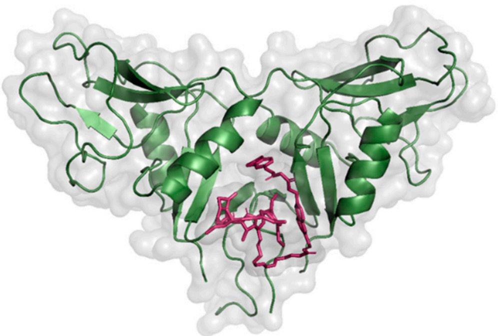

LBE: -7.7 kcal/mol
Pocket volume: 1042 Å^3
Compound **19** (——)

S4. Signs and symptoms recorded from day 1-10 after 17 was injected into the ICR mice

Signs	Negative control		Vehicle control		17 (2.5 mg/kg)			17 (10 mg/kg)		
	**Mice 1**	**Mice 2**	**Mice 1**	**Mice 2**	**Mice 1**	**Mice 2**	**Mice 3**	**Mice 1**	**Mice 2**	**Mice 3**
Stool consistency	N	N	N	N	N	N	N	N	N	N
Mortality	×	×	×	×	×	×	×	×	×	×
Convulsion	×	×	×	×	×	×	×	×	×	×
Aggressiveness	×	×	×	×	×	×	×	×	×	×
Respiration	N	N	N	N	N	N	N	N	N	N
Abnormal activities	×	×	×	×	×	×	×	×	×	×
Skin scar	×	×	×	×	×	×	×	×	×	×
Hair-raised/or not	×	×	×	×	×	×	×	×	×	×
Hair loss	×	×	×	×	×	×	×	×	×	×
Hair shine	×	×	×	×	×	×	×	×	×	×
Others	×	×	×	×	×	×	×	×	×	×

Note: N = Normal, × = Not observed
